# Effects of Combined Low Glutathione with Mild Oxidative and Low Phosphorus Stress on the Metabolism of *Arabidopsis thaliana*

**DOI:** 10.3389/fpls.2017.01464

**Published:** 2017-08-28

**Authors:** Atsushi Fukushima, Mami Iwasa, Ryo Nakabayashi, Makoto Kobayashi, Tomoko Nishizawa, Yozo Okazaki, Kazuki Saito, Miyako Kusano

**Affiliations:** ^1^RIKEN Center for Sustainable Resource Science Yokohama, Japan; ^2^Nissan Chemical Industries, Ltd. Funabashi, Japan; ^3^Graduate School of Pharmaceutical Sciences, Chiba University Chiba, Japan; ^4^Graduate School of Life and Environmental Sciences, University of Tsukuba Tsukuba, Japan

**Keywords:** glutathione, mild abiotic stress, oxidative stress, phosphorus limiting stress, metabolomics, transcriptomics

## Abstract

Plants possess highly sensitive mechanisms that monitor environmental stress levels for a dose-dependent fine-tuning of their growth and development. Differences in plant responses to severe and mild abiotic stresses have been recognized. Although many studies have revealed that glutathione can contribute to plant tolerance to various environmental stresses, little is known about the relationship between glutathione and mild abiotic stress, especially the effect of stress-induced altered glutathione levels on the metabolism. Here, we applied a systems biology approach to identify key pathways involved in the gene-to-metabolite networks perturbed by low glutathione content under mild abiotic stress in *Arabidopsis thaliana*. We used glutathione synthesis mutants (*cad2-1* and *pad2-1*) and plants overexpressing the gene encoding γ-glutamylcysteine synthetase, the first enzyme of the glutathione biosynthetic pathway. The plants were exposed to two mild stress conditions—oxidative stress elicited by methyl viologen and stress induced by the limited availability of phosphate. We observed that the mutants and transgenic plants showed similar shoot growth as that of the wild-type plants under mild abiotic stress. We then selected the synthesis mutants and performed multi-platform metabolomics and microarray experiments to evaluate the possible effects on the overall metabolome and the transcriptome. As a common oxidative stress response, several flavonoids that we assessed showed overaccumulation, whereas the mild phosphate stress resulted in increased levels of specific kaempferol- and quercetin-glycosides. Remarkably, in addition to a significant increased level of sugar, osmolytes, and lipids as mild oxidative stress-responsive metabolites, short-chain aliphatic glucosinolates over-accumulated in the mutants, whereas the level of long-chain aliphatic glucosinolates and specific lipids decreased. Coordinated gene expressions related to glucosinolate and flavonoid biosynthesis also supported the metabolite responses in the *pad2-1* mutant. Our results suggest that glutathione synthesis mutants accelerate transcriptional regulatory networks to control the biosynthetic pathways involved in glutathione-independent scavenging metabolites, and that they might reconfigure the metabolic networks in primary and secondary metabolism, including lipids, glucosinolates, and flavonoids. This work provides a basis for the elucidation of the molecular mechanisms involved in the metabolic and transcriptional regulatory networks in response to combined low glutathione content with mild oxidative and nutrient stress in *A. thaliana*.

## Introduction

Plants can respond to environmental changes and enhance their ability to tolerate biotic and abiotic stresses, including oxidative and nutrient stress (Mittler, 2002, 2006; Vinocur and Altman, 2005; Schachtman and Shin, 2007; Qin et al., 2011). Because reactive oxygen species (ROS) produced via plant metabolism under various stress conditions induce oxidative stress, plants need to reprogram and reconfigure their metabolism to survive (Moller et al., 2007; Baxter et al., 2014). Differences in plant responses to severe and mild abiotic stress have been documented (Kreps et al., 2002; Skirycz et al., 2010; Claeys and Inze, 2013; Dubois et al., 2013, 2015; Claeys et al., 2014). These differences suggest that plants possess highly sensitive systems that monitor environmental stress levels for a dose-dependent fine-tuning of their growth and biological processes in response to stress.

Glutathione is one of the important molecules that protect cells against ROS and maintain intracellular redox homeostasis (Marrs, 1996; Noctor et al., 2012). It has two main stable forms—thiol (GSH) and disulfide (GSSG)—and is synthesized from its constituting amino acids via two steps catalyzed by γ-glutamylcysteine synthetase (*GSH1*) and glutathione synthetase (GSH2) (Dixon et al., 2002). *GSH1* is localized in plastids; GSH2 is primarily localized in the cytosol, but it can also be found in the chloroplast (Wachter et al., 2005). A complete loss-of-function of *GSH1* results in embryo lethality (Cairns et al., 2006), whereas loss-of-function of GSH2 results in a seedling-lethality phenotype (Pasternak et al., 2008). Some mutants with defects in the *GSH1* gene (Cobbett et al., 1998; Vernoux et al., 2000; Ball et al., 2004; Parisy et al., 2007) harbor nucleotide transitions or deletions in a region of the putative catalytic domain of *GSH1* (Parisy et al., 2007). Compared to that in wild-type (WT; Columbia; Col-0) plants, two *GSH1* mutants—*cadmium-sensitive 2-1* (*cad2-1*) (Cobbett et al., 1998) and *phytoalexin-deficient mutant 2-1* (*pad2-1*)—showed lower glutathione content (40 and 22%, respectively) (Parisy et al., 2007). The former mutant was found to be cadmium sensitive (Howden et al., 1995), whereas the latter was highly susceptible to *Pseudomonas syringae* and *Phytophthora brassicae* (Cobbett et al., 1998). The importance of glutathione in plant defense has been well documented (for example, see reviews by Noctor et al., 2012; Queval and Foyer, 2012).

In addition to the above central functions of glutathione, many studies have revealed that glutathione content can contribute to the promotion of tolerance to various environmental stresses (Noctor et al., 1998; Zhu et al., 1999; Gullner et al., 2001; Gomez et al., 2004; Liedschulte et al., 2010). Among the key glutathione-associated genes in *Arabidopsis thaliana*, glutathione-*S*-transferase (GST) plays an important role in the mechanisms underlying plant responses to abiotic stresses, including drought and salt (Ji et al., 2010; Qi et al., 2010; Jha et al., 2011; Xu et al., 2015), cold (Huang et al., 2009), and cadmium (Dixit et al., 2011). Other researchers (Chen et al., 2012; Cheng et al., 2015) determined whether altered glutathione levels affect the abiotic stress tolerance in Arabidopsis and found that an endo- and exogenous increase in glutathione levels in GST-knockout Arabidopsis elicited both drought and salt stress tolerance; *pad2-1* exhibited a survival rate of approximately 50% under stress conditions. In addition to the important role of phytohormones such as salicylic acid (SA), jasmonic acid (JA), and ethylene in plant defense responses, the relationship between glutathione and phytohormones has been considered to play a pivotal role during abiotic stress. Combined transcriptome and proteome analysis of *pad2-1* subjected to combined osmotic and cold stress revealed that glutathione confers stress tolerance to plants via a process associated with lignin, phenylpropanoid, and ethylene biosynthesis (Kumar et al., 2015). Subsequent studies showed that glutathione induces the transcription of genes associated with ethylene biosynthesis in a WRKY33-dependent manner (Datta et al., 2015). Comparative transcriptome and proteome analyses by using ethylene-insensitive, abscisic acid (ABA), and glutathione mutants suggested a crosstalk among ethylene, ABA, and glutathione in inducing stress-responsive genes and proteins to mitigate osmotic and cold stress in Arabidopsis (Kumar et al., 2016). However, little is known about the relationship between glutathione and mild abiotic stress, especially nutrient stress. Furthermore, the effect of stress-induced altered glutathione levels on global primary and secondary metabolism has not been studied.

In this study, we applied a systems biology approach to identify key pathways involved in the gene-to-metabolite networks affected by low glutathione content under mild abiotic stress in Arabidopsis. We used *GSH1* mutants (*cad2-1* and *pad2-1*) and *GSH1*-overexpressing plants and exposed them to two conditions—mild oxidative stress elicited by methyl viologen (MV) and mild stress induced by the limited availability of phosphate (P-lim). We observed no severe visual phenotypes (e.g., chlorosis) in the assayed plants and found that glutathione synthesis mutants and the transgenic plants showed growth similar to that of Col-0 plants under the two mild abiotic stresses. Metabolite and transcript profiling and glutathione quantification showed that the *GSH1* mutants exposed to MV-induced oxidative and P-lim stresses survived by modulating their metabolic and transcriptional networks associated with secondary metabolism.

## Materials and Methods

### Plant Materials and Growth Conditions

Arabidopsis ecotype Columbia (Col-0) was used. The mutant *cad2-1* (Cobbett et al., 1998) was used as an allelic mutant of *pad2-1* (Parisy et al., 2007). Further, *35S::GSH1* transgenic plants, 7-5 and 13-6 (Cheng et al., 2015), were also used to evaluate the shoot phenotypic changes. Plants other than those exposed to MV stress and P-lim were grown in Murashige and Skoog agar medium. Sterilized seeds were stratified at 5°C for 2 days and then sown on Murashige and Skoog medium containing 1% sucrose. Oxidative stress was produced by adding 0.05 μM MV to the Murashige and Skoog medium. The low phosphorus condition was created using P-lim medium in which the phosphate concentration was 20% of that in the Murashige and Skoog medium. Seedlings of Arabidopsis Col-0 and mutants were cultivated in growth chambers at 22°C under 16-h light/8-h dark conditions for 18 days (light strength, 80 μmol⋅m^-2^⋅s^-1^ of the photosynthetic photon flux). GSH1-overexpression lines were cultivated under the same condition for 20 days.

### Metabolite Profiling

Harvested aerial parts of WT and mutant plants (*n* = 8, biological replicates) were frozen immediately in liquid nitrogen to quench enzymatic activity. Primary and secondary metabolites were extracted according to previously established methods; we performed GC-TOF-MS analysis (Kusano et al., 2007a,b) for primary metabolites and LC-q-TOF-MS analysis for secondary metabolites (Matsuda et al., 2009; Nakabayashi et al., 2014) and lipids (Kimbara et al., 2013). The details of metabolite profiling are shown in Supplementary Document S1. Principal component analysis (PCA) was performed using SIMCA-P 12.0 (UmetricsAB^1^) with log_10_ transformation and autoscaling. For stress treatment- and genotype comparison, differentially abundant metabolites were identified using linear regression models in the LIMMA method (Smyth, 2005), which yields false discovery rate (FDR)-adjusted *p*-values for multiple testing problems (Benjamini and Hochberg, 1995). The significance level was set at FDR < 0.05. Our data are reported in a manner compliant with the guidelines recommended by Fernie et al. (2011), as shown in Supplementary Table S4. VENNY^2^ was used to generate the Venn diagram.

### Glutathione Quantification

Plant samples (*n* = 3, biological replicates) were frozen immediately in liquid nitrogen and stored at -80°C until analysis. Each frozen sample was extracted with a 20-fold amount of solvent [methanol/water (8:2 v/v)]. Aerial parts were mashed for 6 min in a 2-mL tube by using a mixer mill (MM400; Retsch, Haan, Germany) at a frequency of 20 Hz. The mixture was centrifuged at 15,000 rpm, and then supernatant containing glutathione was taken out. The supernatant was used for the quantification of GSH and GSSG by using ultra high performance liquid chromatography (UHPLC-MS; Nexera, Shimadzu, Kyoto, Japan) coupled with a triple quadrupole mass spectrometer (TSQ Quantum Ultra Thermo; Fisher Scientific, San Jose, CA, United States). UHPLC separation was performed on an Acquity UPLC BEH C18 column (50 mm × 2.1 mm, 1.7 μm particle size; Waters) maintained at 40°C. Other LC conditions were as follows: flow rate, 0.4 mL/min; injection volume, 5 μL (GSH) and 20 μL (GSSG); solvent system, acetonitrile (0.1% formic acid):water (0.1% formic acid); and gradient program, 2:98 v/v at 0–3 min, 98:2 at 7–10 min. For mass spectrometry (MS) detection, heated-electrospray ionization was used as the ionization source in the positive mode. Other conditions were as follows: sheath gas pressure, 50 arbitrary units; ion sweep cone gas, 0 arbitrary units; vaporizer temperature, 450°C; aux gas pressure, 20 arbitrary units; and spray voltage, 3,000 V. Selected reaction monitoring (SRM) was used to quantify GSH and GSSG. SRM was conducted by scanning the product ions at m/z 162.070 obtained from the fragmentation of the parent ions at m/z 308.167 of GSH. SRM was conducted by scanning the product ions at m/z 231.010 obtained from the fragmentation of the parent ions at m/z 613.244 of GSSG. The collision energy for MS/MS was 27 V. Identities were confirmed by comparing the MS/MS spectra with authentic standards [reduced glutathione (GSH, 97.0%) and oxidized glutathione (GSSG, 98.0%)] purchased from Tokyo Chemical Industry (Tokyo, Japan). Statistical data analysis and plotting were performed using Microsoft Excel and the unpaired Welch’s *t*-test by using the R-function *t.test()*.^3^

### RNA Isolation, Microarray Hybridization, and Data Analysis

We performed analysis of mRNA as described previously (Kusano et al., 2011). Briefly, total RNA was extracted from the 18-day-old aerial part of each mutant and WT sample using the RNeasy plant mini kit (Qiagen^4^) according to the manufacturer’s instructions. Three independent hybridizations were performed using the Affymetrix ATH1 GeneChip microarray, according to the manufacturer’s instructions (Affymetrix).^5^ A single biological replicate was used for each hybridization. Preprocessing and normalization/summarization of all CEL files were performed using R, the Bioconductor (Gentleman et al., 2004), and a robust multi-chip average (RMA) (Bolstad et al., 2003; Irizarry et al., 2003). The quality of the GeneChip data was assessed using the AFFYPLM package (Bolstad et al., 2005). The annotation of each gene in the CSV file ATH1-121501.na31.annot.csv (downloaded in April 2012)^6^ released by Affymetrix was used. For stress treatment- and genotype comparison, differentially expressed genes were detected using linear regression models in the LIMMA method (Smyth, 2005), which provides FDR-adjusted *p*-values for multiple testing problems (Benjamini and Hochberg, 1995). The significance level was set at FDR < 0.05. Genevenn^7^ was used to generate the Venn diagram.

### Functional Enrichment Analysis

The transcript profiles were visualized using MAPMAN (v3.5.1R2) (Thimm et al., 2004) to inspect coordinated changes in metabolism and other biological process. GO enrichment analysis was performed using Cytoscape (v3.2.1) App BiNGO (Maere et al., 2005). The resulting enriched GO categories were visualized using the Enrichment Map (v2.2.0) (Merico et al., 2010; Isserlin et al., 2014) and AutoAnnotate (Kucera et al., 2016).

## Results

### *GSH1* Mutants and *35S::GSH1* Transgenic Plants Showed Similar Growth to That of WT Plants under Mild Abiotic Stress Conditions

**FIGURE 1:**
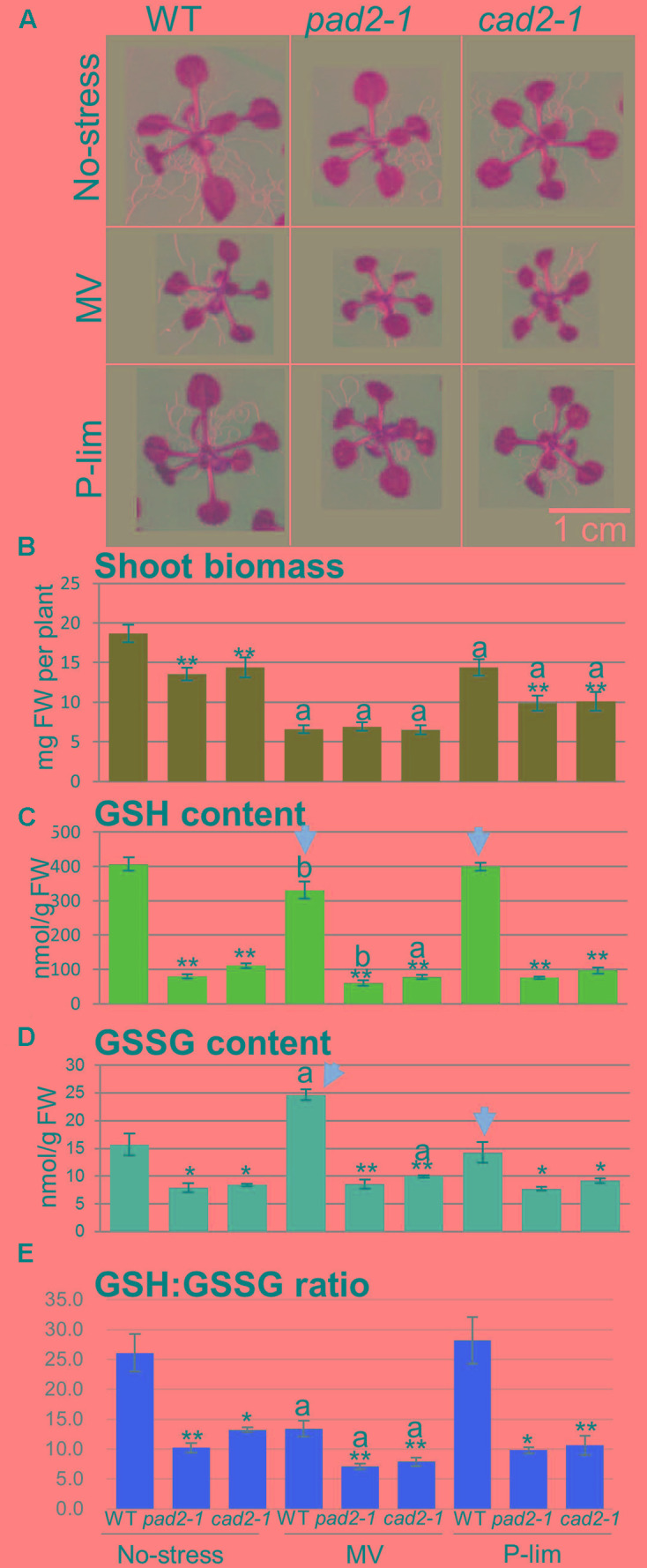
*Visual phenotypes, fresh weight of shoots, and GSH and GSSG levels in* WT plants and the allelic mutants of *GSH1* (*cad2-1* and *pad2-1*) grown under three different conditions. Visible phenotypic changes in 18-day-old *GSH1* mutants grown on Murashige and Skoog medium (no-stress), Murashige and Skoog medium containing 0.05 μM methyl viologen (MV), and low-phosphorus (P-lim) Murashige and Skoog medium **(A)**. Fresh weight (FW) of aerial parts of WT plants and *GSH1* mutants grown under the three conditions (*n* = 20, biological replicates) **(B)**. Quantification of the glutathione content in WT plants and *GSH1* mutants grown under the three growth conditions. Content of the reduced form of glutathione, GSH (*n* = 3, biological replicates) **(C)**. Content of the oxidized form of glutathione, GSSG (*n* = 3, biological replicates) **(D)**. GSH:GSSG ratio as an indirect determinant of oxidative stress **(E)**. Each error bar indicates the standard deviation from the mean. Asterisks represent differences from the control (significant levels were ^∗∗^ α = 0.01 and ^∗^ α = 0.05) by Welch’s *t*-test. The letters “a” and “b” represent significant differences compared to that under the no-stress condition (significant levels were a, α = 0.01 and b, α = 0.05) by Welch’s *t*-test.

Previously, we reported that, under no-stress condition, the shoot phenotype of the *pad2-1* mutant was similar to that of WT plants (background, Col-0), but showed significant changes in the levels of primary metabolites (Fukushima et al., 2014) (**Supplementary Figure S1** and Table S1). To further understand the relationship between the *GSH1* mutation related to glutathione content and mild abiotic stress, we selected two *GSH1* mutants, *pad2-1* and *cad2-1*, and two *GSH1-*overexpressing plants, 7-5 and 13-6 (Cheng et al., 2015). We first assessed the phenotypic changes of individual WT plants and the *GSH1* mutants under different concentrations of MV (**Supplementary Figure S2**). Our current experimental setup was based on these pilot experiments and findings from MV treatment to isolate MV-resistant mutants in the previous studies [for example, see (Fujibe et al., 2004; Sukrong et al., 2012; Egert et al., 2013)]. *GSH1* mutants and *GSH1*-overexpressing plants were grown on untreated medium (control condition), on medium containing a low concentration (0.05 μM) of MV compared to that used in previous studies [e.g., 10 μM MV treatment in AtGenExpress (Kilian et al., 2007)], or on P-lim medium that had a phosphate concentration of 20% of that in the medium (**Supplementary Figure S3**). We evaluated the shoot phenotypic changes in the *GSH1* mutants (**Figure 1**) and *GSH1*-overexpressing plants (**Supplementary Figure S4**). No plants exhibited chlorosis and/or necrosis attributable to treatment with the low MV medium (**Figure 1A** and **Supplementary Figure S4A**). MV treatment of the mutants and WT plants resulted in an approximately 50% growth reduction (**Figure 1B**). The fresh weight of the aerial parts of the mutants grown under control- and P-lim conditions was approximately 70% of that of the WT plants. These findings suggest that, under the no-stress condition, the *GSH1* mutations were silent at the aerial parts, although the mutants were shown to have impaired lateral root density (Schnaubelt et al., 2013, 2015). Therefore, since the applied stress treatments did not result in severe growth inhibition in the assayed plants, we considered them as mild stress treatments (**Figures 1A,B** and **Supplementary Figure S2**).

The GSH and GSSG contents were lower in the mutant than in the WT plants under the no-stress condition (GSH, approximately 20%; GSSG, approximately 50%). Changes in GSH and GSSG contents differed with stress conditions (**Figures 1C,D**). The level of GSH was decreased significantly in WT plants subjected to MV stress, whereas that of GSSG was increased (arrows in **Figures 1C,D**); the GSH and GSSG contents were almost identical under the P-lim and control conditions. The two *GSH1*-overexpressing plants manifested no changes at the phenotypic level under the no-stress condition (**Supplementary Figure S4A**). The fresh weight of the shoot biomass of the *GSH1* mutants, WT plants, and transgenic lines grown under the two mild stress conditions showed similar trends (**Supplementary Figure S4B**). Increased levels of GSH and GSSG failed to rescue the shoot biomass of the *GSH1*-overexpressing plants under the MV condition (**Supplementary Figures S4C,D**). The GSH:GSSG ratio can be regarded as an indirect determinant of oxidative stress; the GSSG level increases and the GSH:GSSG ratio decreases, when the plant cells are exposed to oxidative stress (Queval et al., 2007; Noctor et al., 2012). Under the MV and P-lim conditions, the measured GSH:GSSG ratio was lower in the two *GSH1* mutants than in the WT plants (**Figure 1E**). The GSH:GSSG ratio was largely unaltered in the transgenic lines (**Supplementary Figure S4E**). On the other hand, the overexpression transgenic plants showed high GSH:GSSG ratio under P-lim conditions. Taken together, our findings suggest that, under both mild abiotic conditions, neither low nor high concentrations of glutathione elicited critical changes in the shoot growth and biomass of the examined plants. In our subsequent analysis, we focused on the *GSH1* mutants to avoid pleiotropic effects from *35S*-mediated overexpression.

### Environmental and Genetic Perturbation in the Metabolic Pathways of the *GSH1* Mutants under Mild Abiotic Stresses

**FIGURE 2:**
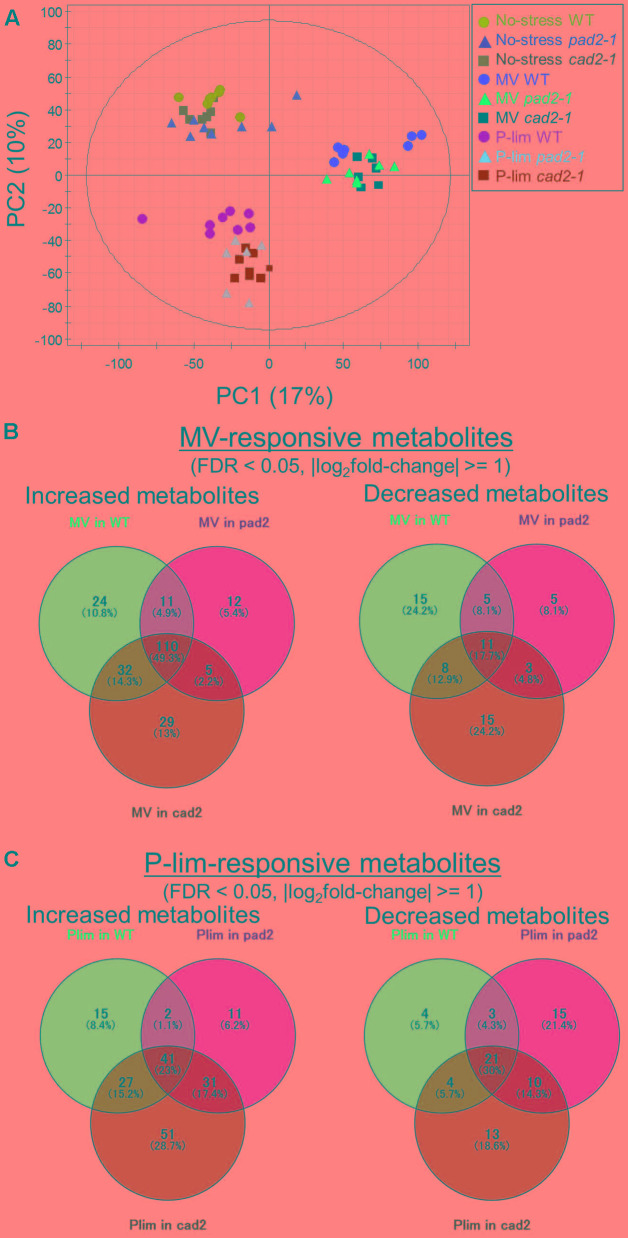
Summary of metabolite profiling conducted in this study. Principal component analysis (PCA) of the metabolite profiles of WT plants and mutants, *cad2-1* and *pad2-1*, exposed to MV and P-lim treatment; integrated data obtained from multi-platform metabolite profiling were used (*n* = 8, biological replicates). The PCA score scatter plot shows that the samples were clustered according to the corresponding abiotic conditions as evidenced by the separation of PC1 **(A)**. Venn diagram of MV- **(B)** and P-lim-responsive (known) metabolites **(C)**. FDR, false discovery rate.

To assess the wide range of metabolic impacts attributed to genotypic differences and types of mild abiotic stresses, we performed multi-platform metabolite profiling (Supplementary Table S2). For a visual inspection of the extent of metabolomic changes under the MV and P-lim stress conditions, we subjected the metabolite profile data to PCA. On the PCA score scatter plot (**Figure 2A**), the samples were clustered according to the applied abiotic stress conditions, as evidenced by the separation of PC1. We observed a genotype-dependent separation in the score scatter plot in the PC1/PC3 or PC2/PC3 direction (**Supplementary Figure S5**). These results indicate that global perturbations due to abiotic stress strongly affected the changes in the metabolite level of the mutant and WT plants.

We compared the metabolic responses in the metabolite profiles of the WT plants and *GSH1* mutants grown under the no-stress condition or exposed to MV or P-lim stress. Under MV-induced oxidative stress, a total of 285 significantly changed metabolites was identified (**Figure 2B**), of which 223 were increased and 62 were decreased, respectively, including the commonly and exclusively accumulated metabolites. In all, 110 metabolite levels increased in the WT plant and *GSH1* mutants after MV treatment, whereas 11 metabolite levels decreased (**Figure 2B**). For P-lim, we identified 178 increased and 70 decreased metabolites, respectively. The levels of 41 metabolites increased in the 3 genotypes under P-lim condition, whereas 21 metabolite levels decreased (**Figure 2C**). The number of significantly altered metabolites between *GSH1* mutants and WT plants under mild abiotic stress was smaller than that of the treatment (**Supplementary Figure S6**), indicating consistency with the results of PCA score plots.

#### MV-Responsive Metabolites

**FIGURE 3:**
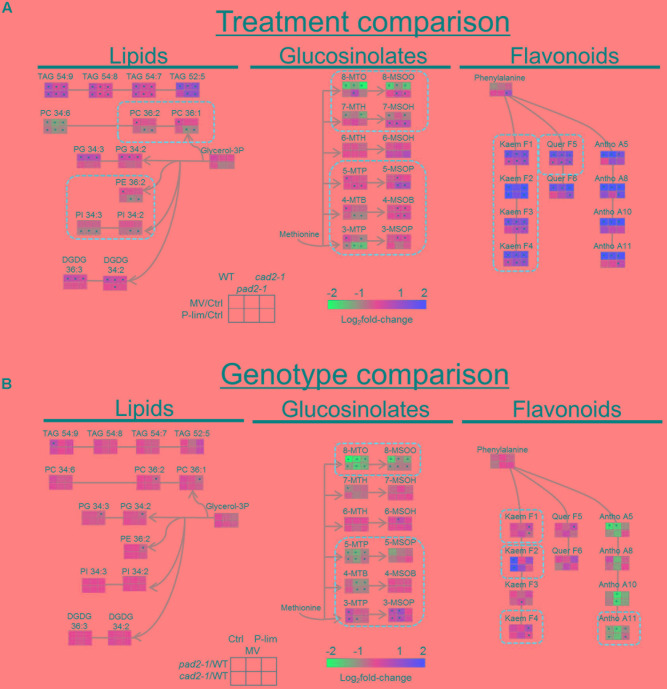
Changes in metabolite levels associated with secondary metabolism in the *GSH1* mutants and WT after MV and P-lim treatments. **(A)** Stress treatment comparison: Log_2_fold-change in metabolites exposed to the two abiotic stresses. The fold-change is represented by two directed colors; red and blue indicate increase and decrease, respectively, in the metabolite level elicited by MV treatment or P-lim. **(B)** Genotype-dependent comparison: Red and blue indicate increase and decrease, respectively, in the metabolite levels in the mutants vs. WT plants. For abbreviations of the metabolite names, see Supplementary Table S5. *n* = 8, biological replicates, ^∗^FDR < 0.05.

The differentially accumulated metabolites involved in primary and secondary metabolism were visualized on a simplified metabolic map (**Supplementary Figure S7**). Stress-specific metabolite alterations against oxidative stress were significantly increased, for example, γ-amino butyrate (GABA), 2-oxo glutarate, proline, and putrescine. MV treatment resulted in a significant increase in the level of sugar and osmolytes. Sucrose, glucose, fructose, xylose, maltose, arabinose, trehalose, raffinose, inositol, and galactinol were observed in all the samples (**Supplementary Figure S7A**). Notable changes were observed in metabolite levels associated with secondary metabolism in the *GSH1* mutants and WT plants by MV and P-lim treatments (**Figure 3**). Triacylglycerols (TAG) increased under almost all conditions, whereas phosphatidylcholine (PC) 34:6 decreased under all conditions. The phospholipids, 34:2 and 34:3 phosphatidylglycerols (PG) and 34:2 and 36:3 digalactosyldiacylglycerols (DGDG), increased after MV treatment. As for glucosinolates, the level of long-chain aliphatic glucosinolates [8-MTO (8-methylthio-*n*-octylglucosinolate) and 8-MSOO (8-methylsulfinyl-*n*-octylglucosinolate)] was remarkably decreased after MV treatment. Antioxidants, including flavonols and anthocyanins except kaempferol (F2), were significantly increased across the *GSH1* mutants after MV treatment.

#### Low Phosphate-Responsive Metabolites

In addition to lowered glucose-6-phosphate and fructose-6-phosphate levels under P-lim condition in both the WT and the mutants, several low phosphate-responsive meta-bolites were identified. Examples include the phospholipids, 36:1 and 36:2 PCs, 36:2 phosphatidylethanolamine (PE), and 34:2 and 34:3 phosphatidylinositol (PI), decreased in the *GSH1* mutants under P-lim condition. In the glucosinolate biosynthesis, the level of aliphatic glucosinolates, 4-MSOB (4-methylsulfinyl-*n*-butylglucosinolate), 5-MTP (5-methylthio-*n*-pentylglucosinolate), 5-MSOP (5-methylsulfinyl-*n*-pentylglucosinolate), 7-MSOH (7-methylsulfinyl-*n*-heptylglucosinolate), and 8-MSOO (8-methylsulfinyl-*n*-octylglucosinolate), increased in the *GSH1* mutants under P-lim condition (**Figure 3A**). In contrast, P-lim stress elicited a significant decrease in 3-MTP (3-methylthio-*n*-propylglucosinolate) in all genotypes/conditions. P-lim stress also resulted in moderate increases of detected flavonoids with kaempferol aglycone (F1, F2, F3, and F4) and that of quercetin (F5) in the *GSH1* mutants. There was also opposite changes in dihydrouracil levels in a genotype/stress dependent manner. Other metabolites such as the monosaccharides xylose and arabinose and the disaccharide maltose, tryptophan, octadecatrienoate, fumarate, and threonine were also increased under P-lim condition (**Supplementary Figure S7A**).

#### *GSH1* Mutant-Specific Changes in Metabolite Levels under Mild Abiotic Stress

A genotype-dependent comparison of the metabolite profiles of WT plants and *GSH1* mutants grown under abiotic stress conditions is shown in **Figure 3B**. Compared to that of WT plants, the metabolite profile of each mutant allele under both stress conditions exhibited a similar trend in metabolic responses. Metabolite changes in the biosynthetic pathways of glucosinolates and anthocyanins in the *pad2-1* and *cad2-1* mutants were noted; the flavonol level tended to increase and the level of anthocyanins was remarkably decreased under both the stress conditions. The level of low-phosphate responsive kaempferol-type flavonols, F1 and F4, was higher in the *GSH1* mutants than in the WT plants under P-lim condition. In the mutants subjected to no-stress and MV conditions, short-chain glucosinolates, 3-MTP and 3-MSOP (3-methylsulfinyl-*n*-propylglucosinolate), were increased and long-chain glucosinolates, 8-MTO and 8-MSOO, were decreased. The level of metabolites [e.g., 8-MSOO and A11, one of the major anthocyanin that contains three acyl groups, namely cyanidin-3-*O*-[2-*O*-(2-*O*-(sinapoyl)-xylosyl)-6-*O*-(*p-O*-coumaroyl)-glucoside]-5-*O*-[6-*O*-(malonyl)-glucoside (Tohge et al., 2005)] downstream of glucosinolate and anthocyanin pathways was decreased.

### Genome-Wide Expression Changes in Response to Mild Abiotic Stress

#### Functional Enrichment Analysis

**FIGURE 4:**
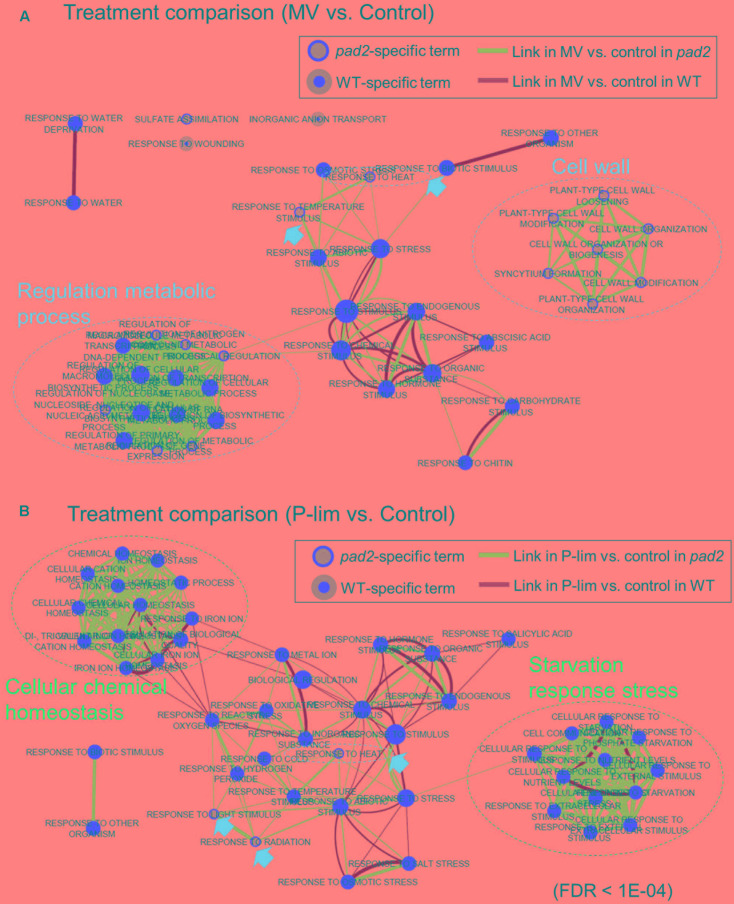
Enrichment map showing biological processes between stress treatment and no-stress condition based on hypergeometric tests of gene ontology (GO) terms by using BiNGO software (Maere et al., 2005). The map shows the enriched GO terms in MV-treated vs. no-stress condition **(A)** and in P-lim vs. no-stress **(B)**. Enrichment map can be used to compare differential transcriptomic responses to abiotic stress between 2 genotypes (i.e., *pad2-1* vs. WT). Nodes represent GO terms, and links between nodes represent gene overlap between GO terms, resulting in a rapid identification of the major enriched functional categories. Inner circle size of each node represents the number of DEGs in “comparison 1” (e.g., MV vs. no-stress in WT) within the GO term in a biological process. Node border size represents the number of DEGs in “comparison 2” (e.g., MV vs. no-stress in *pad2*) within the GO term in a biological process. Color of the node and border indicate significance based on the BiNGO FDR of the GO term for “comparison 1” and “comparison 2,” respectively. The red-filled nodes highlight the major GO functional terms. Link size shows the number of DEGs that overlap between the two connected GO terms (Jaccard coefficient, the cut-off is 0.25). Green links correspond to both datasets when it is the only colored link. Green links indicate “comparison 1” and blue indicates “comparison 2.” Blue dotted circles represent summarized GO term clusters based on AutoAnnotate (Kucera et al., 2016). The maps were generated using Cytoscape (v3.2.1) Enrichment Map plugin (Merico et al., 2010; Isserlin et al., 2014).

For a better understanding of the metabolic responses to the imposed stress conditions, we performed transcript profiling by using microarray analysis. In this experiment, we focused on *pad2-1* as a representative *GSH1* allele in transcript profiling (Supplementary Table S3). To characterize the gene expression patterns of differentially expressed genes (DEGs) in the WT and *pad2-1* plants subjected to MV or P-lim stress conditions, we performed gene ontology (GO) enrichment analysis. By using Enrichment Map (Merico et al., 2010; Isserlin et al., 2014), we revealed biological processes between stress treatment and no-stress condition based on hypergeometric tests of GO terms (**Figure 4**). The enriched GO terms in MV-treated vs. no-stress condition for each genotype are shown in **Figure 4A**. We also created an enrichment map showing biological processes between *pad2* and WT under two differential conditions based on hypergeometric tests of GO term enrichment analysis (**Supplementary Figure S8**). We identified GO terms “sulfate assimilation,” “response to heat,” and “response to temperature stimulus” as well as a cluster-comprising terms related to “cell wall” and partially related to “regulation metabolic process” as *pad2-1*-specific expression responses to MV treatment (**Figure 4A**). For the enriched GO terms in P-lim vs. no-stress, GO terms “response to heat,” “response to light stimulus,” and “response to radiation” were enriched in only *pad2-1* (**Figure 4B**). To inspect the expression level of genes in the primary and secondary metabolism, we also performed MAPMAN (Thimm et al., 2004) analysis. For metabolism, remarkable changes were noted for many transcripts directly or indirectly involved in hormone, secondary, and cell wall metabolism (**Supplementary Figures S9**–**S11**). Coordinated induction of genes for ABA and JA metabolism was noted in the P-lim treated *pad2-1*. For secondary metabolism, we identified coordinated expression for glucosinolate metabolism as *pad2-1*-specific response to both mild abiotic stresses. In addition, there were coordinated changes in transcript levels associated with phenylpropanoids and phenolics as a *pad2-1*-specific response to P-lim. Cell wall modification and pectin esterases were identified as coordinated pathways in only WT plants under P-lim condition.

#### Mild Oxidative- and Low Phosphate-responsive Genes

**FIGURE 5:**
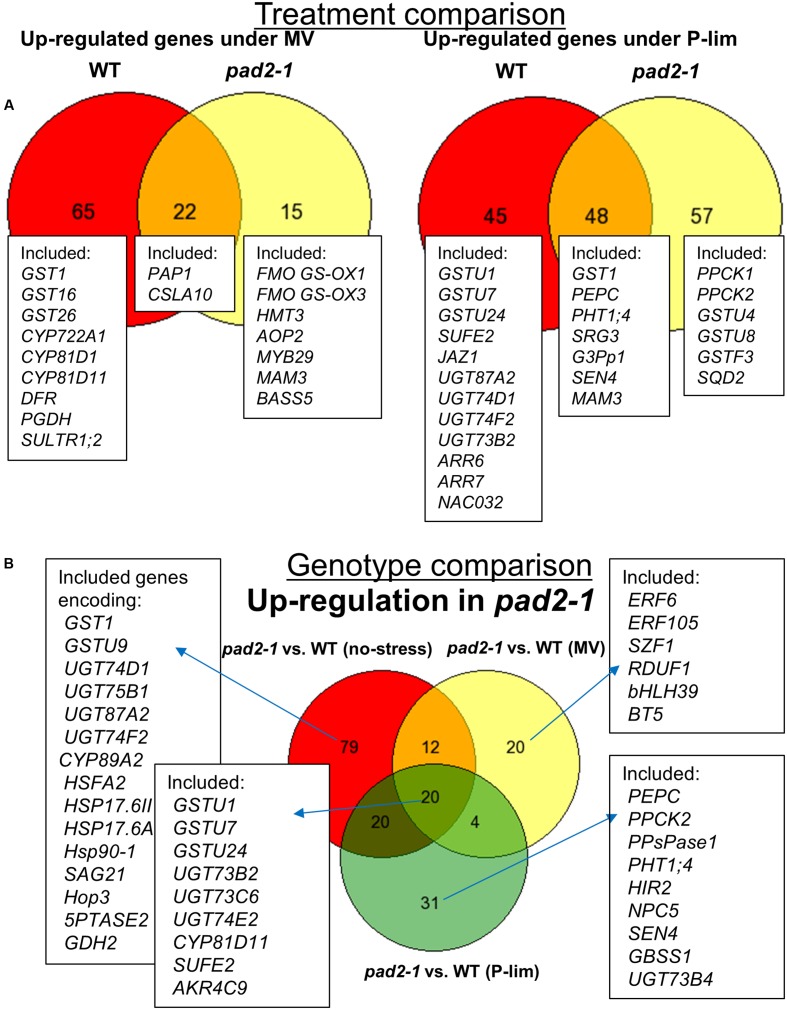
Venn diagrams representing the classification of genes based on microarrays. **(A)** We identified 87 MV-inducible genes in WT plants and 37 MV-inducible genes in the *pad2-1* mutant. We also identified 93 low-P-inducible and 105 low-P-inducible genes in WT plants and the *pad2-1* mutant, respectively. **(B)** Genotype-dependent up-regulated genes. Under the control condition, MV-treatment, and low P stress condition, we identified 131, 56, and 75 up-regulated genes, respectively, in *pad2-1*. We used genevenn (http://genevenn.sourceforge.net/vennresults.php) to draw the diagrams. Differentially expressed genes were identified using a threshold |log_2_ fold-change| ≥ 1 and FDR < 0.05.

Methyl viologen treatment resulted in 87 (WT plants) and 37 (*pad2-1*) MV-inducible genes (**Figure 5A**, left). Genes down-regulated by genetic and environmental perturbations are shown in Venn diagrams that represent the classification of genes (**Supplementary Figure S12**). The stress-inducible genes included those encoding GST, UDP-glycosyl transferase (UGT), and cytochrome P450 (CYP), which are associated with detoxification and transcriptome responses when plants are exposed to oxidative stress (Dixon et al., 2002). In addition, genes related to flavonoids [e.g., *PRODUCTION OF ANTHOCYANIN PIGMENT 1* (*PAP1*)] and glucosinolates [e.g., *MYB DOMAIN PROTEIN 29* (*MYB29*)/*PRODUCTION OF METHIONINE-DERIVED GLUCOSINOLATE 2* (*PMG2*)] were up-regulated.

P-lim condition resulted in 93 (WT) and 105 (*pad2-1*) low phosphorus-inducible genes (**Figure 5A**, right). The inducible genes included *SPX1*, phosphate starvation-induced genes (*PS2* and *PS3*), *phosphate transporter 1;4* (*PHT1;4*), and *PHOSPHOCHOLINE PHOSPHATASE 1* (*PEPC1*), which are known to be up-regulated during phosphorus deprivation (Morcuende et al., 2007). As *pad2-1*-specific transcriptional responses, genes encoding phosphoenolpyruvate carboxylase kinase (PPCK1 and PPCK2), glutathione transferases (e.g., GSTU4, GSTU8, GSTF3), and lipid biosynthesis enzymes [e.g., SULFOQUINOVOSYLDIACYLGLYCEROL 2 (SQD2)] were up-regulated.

We also identified genotype-dependent altered genes. Under the no-stress condition, 131 up-regulated genes were identified in *pad2-1*; their number was 56 after MV treatment and 75 after exposure to low P-stress (**Figure 5B**). Several *pad2-1*-specific expression changes were noted under the different environmental conditions in genes encoding GSTs, UGTs, CYPs, heat shock proteins (HSPs), senescence-associated genes (SAGs), and transcription factors such as ETHYLENE RESPONSIVE ELEMENT BINDING FACTOR 6 (ERF6) and HEAT SHOCK TRANSCRIPTION FACTOR A2 (HSFA2).

### Transcript Changes in Mild Stress-Responsive Genes and Well-Characterized Stress-Responsive Factors

Among the DEGs, we focused on “marker genes” that are highly induced by stress treatments (Claeys et al., 2014). They include marker genes elicited under oxidative stress [*NAC DOMAIN-CONTAINING PROTEIN32* (*NAC032*) and *ALDO-KETO REDUCTASE 4C9* (*AKR4C9*)], those induced by abscisic acid (ABA) [*NINE-CIS-EPOXYCAROTENOID DIOXYGENASE3* (*NCED3*) and *CYP707A3*] and dehydration [*LATE EMBRYOGENESIS ABUNDANT-LIKE5* (*LEA5*), also known as *SAG21*], mild osmotic stress markers [*MYB51*, *ERF5*, and *WRKY DNA-BINDING PROTEIN33* (*WRKY33*)], and genes overexpressed in response to phosphate starvation [*SQD1* and *SQD2*] (**Table 1**). We found that the expression of the oxidative stress markers *NAC032* and *AKR4C9* was remarkably up-regulated in *pad2-1* grown under MV stress and control conditions. Under P-lim stress, *NAC032* and *AKR4C9* genes were up-regulated in WT plants; genotype comparison showed the up-regulation of the *AKR4C9* gene in *pad2-1* plants. Marker gene expressions associated with ABA and mild osmotic stress were largely unaltered except for *LEA5*/*SAG21* (up-regulated in *pad2-1* under control conditions) and *WRKY33* (down-regulated in *pad2-1* by MV treatment). With respect to stress marker responses to phosphate starvation, both *SQD1* and *SQD2* genes were up-regulated in *pad2-1* under P-lim stress; the *SQD1* gene was only up-regulated in WT plants.

**Table 1:** Transcriptional changes in well-characterized stress-responsive factors in Arabidopsis.

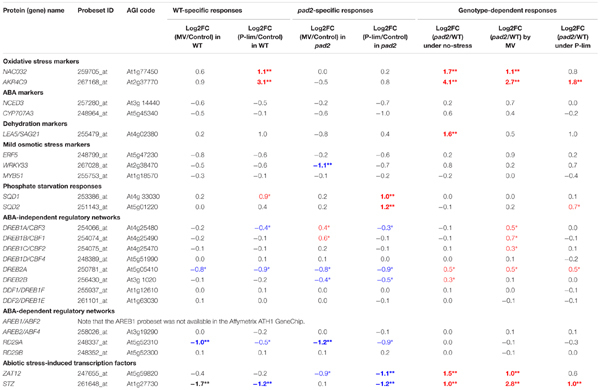

*A single asterisk (^∗^) indicates significance (FDR < 0.05). Bold numbers with double asterisks (^∗∗^) identify a |log_*2*_ fold-change|≥ 1.0 and FDR < 0.05.*

To assess the changes in other stress-responsive genes, including well-known transcription factors, in the WT plants and the *GSH1* mutants exposed to abiotic stress, we investigated the general patterns of gene expression associated with DREB/CBF (dehydration-responsive-element binding protein/C-repeat-binding factor) and AREB/ABF (ABA-responsive element-binding/ABA-responsive element-binding factor) protein. They play a crucial role in the adaptation to various stresses (Yamaguchi-Shinozaki and Shinozaki, 2006). Among *DREB*/*CBF* and *AREB*/*ABF* genes, the transcript level of *DREB2A* was down-regulated (log_2_ fold-change, approximately -1) in the WT and *pad2-1* plants grown under both stress conditions. The expression of *DREB2A* was gradually induced by H_2_O_2_ (Sakuma et al., 2006). Under the MV condition, the mutants showed slight up-regulation of *DREB1A/CBF3*, *DREB1C/CBF2*, and *DREB2A*. The expression of *AREB2/ABF4* in the WT and *pad2-1* plants was unchanged under the three growth conditions. An abiotic stress-responsive gene in Arabidopsis is *Responsive to Desiccation* (*RD*) (Yamaguchi-Shinozaki and Shinozaki, 1993). Both WT and *pad2-1* plants showed down-regulation of *RD29A* under both stress conditions; the expression of *RD29B* in WT and *pad2-1* plants was unaltered under the three conditions (**Table 1**). The expression of two genes encoding ZAT12 (Davletova et al., 2005; Vogel et al., 2005) and STZ (Sakamoto et al., 2004) was markedly higher in the MV-treated *pad2-1* than in WT plants (**Table 1**).

### Integrated Pathway Analysis Reveals Specific Changes in the Biosynthesis of Aliphatic Glucosinolates and Anthocyanins at the Metabolite and Transcript Levels in Mild Stress-Treated *pad2-1* Mutants

**FIGURE 6:**
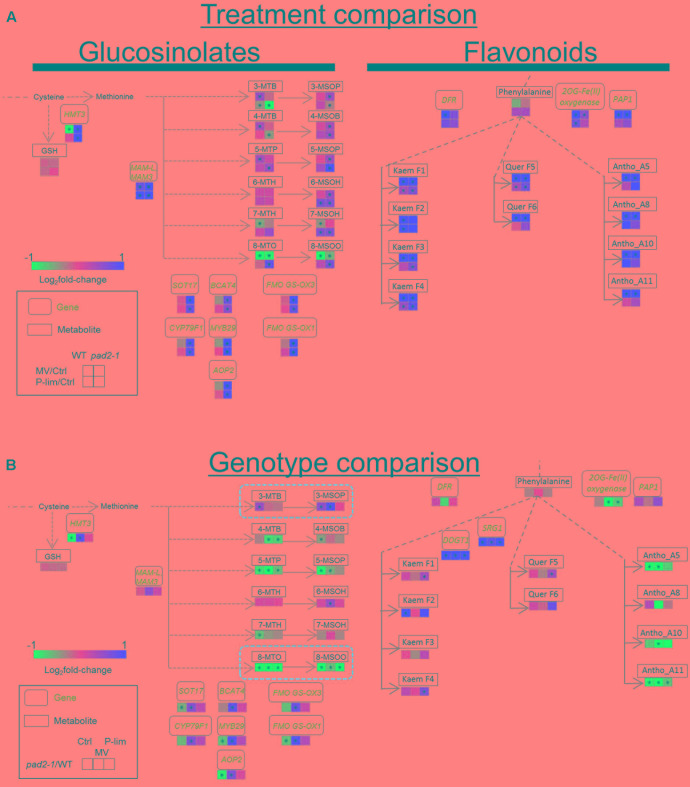
Integrated metabolomic and transcriptomic responses in the glucosinolate and flavonoid pathways of WT plants and *pad2-1* mutant exposed to MV and P-lim stress. **(A)** Stress-treatment comparison: The log_2_fold-change in the metabolomic/transcriptomic levels elicited by exposure to MV or P-lim. Red indicates an increase (up-regulation) by MV or P-lim and blue indicates decrease (down-regulation) in the *pad2-1* mutant compared to that in WT plants. **(B)** Genotype- dependent comparison: Red and blue indicate an increase and a decrease, respectively, in the metabolite and transcript levels in the mutant compared to that in WT plants. ^∗^FDR < 0.05. Abbreviations: Ctrl, control (no-stress) condition; HMT3, homocysteine *S*-methyltransferase; MAM3/MAM-L, methylthioalkylmalate 3/methylthioalkylmalate-L; SOT17, sulfotransferase 17; BCAT4, branched chain aminotransferase 4; FMO GS-OX3, flavin-monooxygenase glucosinolate *S*-oxygenase 3; CYP79F1, cytochrome P450 CYP79F1; MYB29, MYB DOMAIN PROTEIN 29; FMO GS-OX1, flavin-containing monooxygenase glucosinolate *S*-oxygenase 1; AOP2, alkenyl hydroxialkyl producing 2; DFR, dihydroflavonol 4-reductase; 2OG-Fe(II) oxygenase, 2-(oxo)glutarate and Fe(II)-dependent oxygenase; PAP1, production of anthocyanin pigments 1; SRG1, SENESCENCE-RELATED GENE 1; DOGT1, DON-GLUCOSYLTRANSFERASE 1; Kaem F1, Kaempferol- 3,7-*O*-di-rhamnopyranosid; Kaem F2, kaempferol-3-*O*-beta-glucopyranosyl-7-*O*-alpha-rhamnopyranoside; Kaem F3, kaempferol-3-*O*-alpha-*L*-rhamnopyranosyl (1→2)-beta-*D*-glucopyranoside-7-*O*-alpha-*L*-rhamnopyranoside; Kaem F4, kaempferol-3-Galactoside-6″-Rhamnoside-3′′′′-Rhamnoside; Quer F5, quercetin-3,7-*O*-alpha-*L*-dirhamnopyranoside; Quer F6, quercetin-3-*O*-beta-glucopyranosyl-7-*O*-alpha-rhamnopyranoside; Antho A5, cyanidin conjugate (A5); Antho A8, cyanidin conjugate (A8); Antho A10, cyanidin conjugate (A10); Antho A11, cyanidin conjugate (A11); 8-MTO, 8-methylthio-*n*-octylglucosinolate; 7-MTH, 7-methylthio-*n*-heptylglucosinolate; 6-MTH, 6-methylthio-*n*-hexylglucosinolate; 5-MTP, 5-methylthio-*n*-pentylglucosinolate; 4-MTB, 4-methylthio-*n*- butylglucosinolate; 3-MTP, 3-methylthio-*n*-propylglucosinolate; 8-MSOO, 8-methylsulfinyl-*n*-octylglucosinolate; 7-MSOH, 7-methylsulfinyl-*n*-heptylglucosinolate; 6-MSOH, 6-methylsulfinyl-*n*-hexylglucosinolate; 5-MSOP, 5-methylsulfinyl-*n*-pentylglucosinolate; 4-MSOB, 4-methylsulfinyl-*n*-butylglucosinolate; 3-MSOP, 3-methylsulfinyl-*n*-propylglucosinolate. See also Supplementary Table S5.

To assess the coordinated responses of glucosinolate and flavonoid biosynthesis under mild stress conditions, we performed pathway analysis of combined metabolite and transcript profile data. The integrated metabolomic and transcriptomic responses in WT and *pad2-1* plants under the two stress conditions are shown in **Figure 6**. As *pad2-1*-specific metabolite responses, the level of short-chain aliphatic methylsulphinylalkyl glucosinolates [4-MSOB, and 5-MSOP] and 6-MSOH (6-methylsulfinyl-*n*-hexylglucosinolate) was increased in *pad2-1* under both stress conditions; the difference was a significant but moderate effect size (all the ranges from 0.39 to 0.97 in log_2_ fold-change) (**Figure 6A**, left). The level of 8-MTO and 8-MSOO was decreased by MV treatment, irrespective of the genotype (all the ranges from -1.37 to -0.56 in log_2_ fold-change). The level of 5-MTP, 7-MSOH, 8-MTO, and 8-MSOO was increased in *pad2-1* under P-lim condition (all the ranges from 0.45 to 0.82 in log_2_ fold-change). Gene expressions associated with glucosinolate biosynthesis were up-regulated in *pad2-1* grown under abiotic stress conditions. These included some genes encoding *methylthioalkylmalate synthase* (*MAM*) (Textor et al., 2007), *MYB29* (Hirai et al., 2007; Sonderby et al., 2007; Gigolashvili et al., 2008), *branched chain aminotransferase* (*BCAT*) (Schuster et al., 2006), *flavin-containing monooxygenase* (*FMO*) (Hansen et al., 2007), and other genes involved in glucosinolate biosynthesis. Under the MV condition, the transcript levels of these genes were significantly higher in *pad2-1* mutants than in WT plants, except for the *MAM* gene (**Figure 6B**, left). Compared to that in WT plants, the level of 8-MSOO was decreased in *pad2-1* mutants under all conditions, whereas that of 3-MSOP was increased under no-stress and MV conditions.

In WT and *pad2-1* plants, MV treatment triggered the up-regulation of the transcript levels of *dihydroflavonol reductase (DFR)*, *2OG-Fe(II) oxygenase*, and *PAP1* after a remarkable increase in flavonols and anthocyanins (**Figure 6A**, right). As discussed previously (Saito et al., 2008; Fukushima and Kusano, 2014; Nakabayashi and Saito, 2015), the metabolomic and transcriptomic responses in the anthocyanin pathway in Arabidopsis were well correlated under oxidative stress conditions. In contrast to mild MV-induced oxidative stress, the increased level of kaempferols mentioned above was not correlated with the transcript levels in the pathway under P-lim condition. Compared to that in WT plants, *pad2-1* mutants under MV and P-lim conditions showed a significant increase in two genes encoding SENESCENCE-RELATED GENE 1 (SRG1) and DON-GLUCOSYLTRANSFERASE 1 (DOGT1) in the flavonol biosynthetic pathway; the level of flavonols was largely unaltered (**Figure 6B**, right). When we focused on genotype-dependent changes, we found that, in the anthocyanin synthetic pathway, a remarkable decrease in the transcript level of *2OG-Fe(II) oxygenase* and the anthocyanin level in *pad2-1* mutant was noted compared to that in WT plants. Our finding that the anthocyanin level was lower in the mutant was consistent with that of previous findings on *GSH1*-antisense transgenic plants reported by (Xiang et al., 2001).

## Discussion

### Impact of Low Glutathione Content and Visual Phenotypes of *GSH1* Mutants and Transgenic Plants

In this study, we investigated the changes in metabolite and transcript levels that reflect regulatory networks compensating for the reduction in the glutathione levels to enable the survival of *GSH1* mutants under two mild abiotic stress conditions. We chose MV because the reduction of GSSG to GSH plays an important role in maintaining and regulating the cellular redox status during oxidative stress induced by MV. *GSH1* is localized in plastids (Wachter et al., 2005), and MV is a fairly specific stimulus for ROS production in the chloroplast (Noctor et al., 2015). We chose the P-lim condition because phosphorus is an important component of the phospholipid membrane in the chloroplast (Hartel et al., 2000; Andersson et al., 2003; Jouhet et al., 2004; Okazaki et al., 2013). While phosphorus is also involved in plant growth and metabolism as one of the major nutrient ions, the molecular basis of plant adaptation to deal with a low phosphorus environment has not been completely elucidated. Although the series of genes and enzymes that are associated with these plant responses and adaptations (*GSH1* is an example) are known, the comprehensive molecular mechanisms of their regulation and control are less well understood. Therefore, we applied the integrated omic approach by using high-throughput molecular phenotyping to *GSH1* mutants grown under mild MV and P-lim conditions.

*GSH1* mutants, *cad2-1* (Cobbett et al., 1998) and *pad2-1* (Parisy et al., 2007), showed differences in sensitivity to cadmium and biotic stresses from that of WT plants (Bednarek et al., 2009; Clay et al., 2009; Schlaeppi et al., 2010). Generally, glutathione synthesis mutants show shorter primary roots and lower levels of lateral root density compared to that in WT plants because of glutathione depletion (Schnaubelt et al., 2015). Low glutathione levels do not largely affect the shoot phenotypes of glutathione synthesis mutants under no-stress conditions. The characterization of leaf area phenotypes by using a high-resolution phenomic approach showed that glutathione synthesis mutants, except for an allele of *GSH1* mutant called *regulator of APX2 1-1* (*rax1-1*) (Ball et al., 2004), were not as sensitive as the WT plants under 1 μM MV treatment (Schnaubelt et al., 2013). We found that the two *GSH1* mutants (*cad2-1* and *pad2-1*), *GSH1*-overexpressing plants [7-5 and 13-6 (Cheng et al., 2015)], and WT plants exhibited a similar level of growth reduction under the mild MV stress condition (**Figure 1** and **Supplementary Figure S4**). This suggests that neither a low nor high content of glutathione can compensate for the reduction in the shoot biomass of Arabidopsis under MV treatment. In WT plants, the level of GSH was slightly lower in the MV than in the no-stress condition and the GSSG level was increased (**Figures 1C,D**). The GSH:GSSG ratio in WT plants was similar under the control and P-lim condition, suggesting that the oxidative stress status was low in both (**Figure 1E**). Since glutathione peroxidase catalyzes the changes from GSH to GSSG, tracing the GSH:GSSG ratio by measuring GSH and GSSG might yield a specific marker of oxidative stress, e.g., increased H_2_O_2_ metabolism (Queval et al., 2007; Noctor et al., 2012).

### Effects of Combined Low Glutathione and Mild Abiotic Stress on Metabolism in Arabidopsis

Our metabolite and transcript profiling revealed distinctive metabolic and transcriptional responses against two mild abiotic stresses. Genetic perturbation of metabolic responses in the WT plants and the two allelic mutants subjected to oxidative stress by MV clearly showed a specific enhancement in metabolite production in the sugar and flavonoid metabolism (**Figure 3** and **Supplementary Figure S7**). This type of stress might result in an altered metabolic status to compensate for the reduced ROS scavenging ability by reprogramming the sugar and flavonoid metabolism to produce specific antioxidant metabolites that consist of aglycone with sugar conjugates, i.e., flavonols and anthocyanins, as a common oxidative stress response. We observed that our P-lim condition resulted in increased levels of specific flavonol-glycosides, kaempferol 3-*O*-rhamnoside-7-*O*-rhamnoside (F1), kaempferol 3-*O*-[glucosyl(1 → 6)glucoside] 7-*O*-rhamnoside (F4), and quercetin 3-*O*-rhamnoside-7-*O*-rhamnosides (F5). Previous transcript- and flavonoid profiling demonstrated that the *LATERAL ORGAN BOUNDARY DOMAIN* (*LBD*) gene family of transcription factors (*LBD37*, *LBD38*, and *LBD39*) regulate the late steps of anthocyanin-specific biosynthesis and flavonol glycosides [F1 to F6, see (Tohge et al., 2005)] derived from kaempferol or quercetin were largely unaltered in the *LBD*-overexpression or mutant seedlings irrespective of nitrogen supply (Rubin et al., 2009). Another report also demonstrated that flavonoid overaccumulation [e.g., flavonols (F1, F2, F3, F5, and F8) and anthocyanin] was important to enhanced tolerance to oxidative- and drought stresses (Nakabayashi et al., 2014). Together these observations suggest that specific flavonols and anthocyanins play a distinct role in mitigating abiotic stress in Arabidopsis.

Changes in the GSH level under each stress condition (MV or P-lim vs. control) were almost identical in WT plants and the mutants (**Figure 1** and **Supplementary Figure S7**). Antioxidants such as ascorbate and glutathione play a central role in plant defense under oxidative stress conditions (for example, see Foyer and Noctor, 2009). Conversely, phytochelatins, polymerized to form GSH, protect plants from heavy metal toxicity (Xiang et al., 2001; Schat et al., 2002). In general, GSH is maintained at a high concentration in the sulfur assimilation pathway (Saito, 2000, 2004). Glucosinolates and phytochelatins are downstream metabolites in the pathway. No accumulation of phytochelatins was noted in the *cad2-1* mutant (Jozefczak et al., 2015). Our metabolite profiling showed that, with the exception of 8-MTO and 8-MSOO, the level of glucosinolates was higher under both stress conditions than under no-stress in *pad2-1* and *cad2-1* mutants (**Figures 3A**, **5A**, left). The accumulation patterns of glucosinolates under the applied stress conditions might be explained by metabolic perturbation resulting in a decrease of phytochelatin and an increase of glucosinolate levels to compensate for the reduced glutathione level against mild oxidative stress. Glucosinolates might protect against oxidative stress in the *GSH1* mutants.

As mentioned above, our results imply that coordinated responses of glucosinolate and flavonoid biosynthesis at the metabolite and transcript levels are associated with the plant response to MV-induced oxidative stress and to stress elicited by P-lim (**Figure 6**). We found that, in the *GSH1* mutants, a short-chain aliphatic glucosinolate (3-MSOP) was over-accumulated under mild oxidative stress, and the level of long-chain aliphatic glucosinolates was decreased (**Figures 3B**, **6B**, left). Glucosinolate biosynthesis involves the breakdown of methionine to produce short-chain glucosinolates that harbor three carbons in their moiety (3C-glucosinolates) in the first round of glucosinolate chain elongation. Subsequently, 4C- and long-chain glucosinolates are synthesized by further rounds of elongation (Field et al., 2004). These elongation reactions are mediated by genes depicted in **Figure 6** (left). The increase in the 3-MSOP level together with a decrease in the level of long-chain glucosinolates might be due to the gap and could result in the production of glucosinolates exhibiting different chain lengths. By using the insect herbivore *Spodoptera littoralis*, Schlaeppi et al. (2008) found that the *pad2-1* mutant exhibited a reduction of two 3C- and 4C-glucosinolates, 4-methylsulfinylbutylglucosinolate and indolyl-3-methylglucosinolate, and that their reduction was correlated with the reduced GSH level (approximately 20% of that in WT plants). In addition, glutathione has possible roles as an s atom donor for glucosinolates (Noctor et al., 2012). It could be a reason of the decrease of long-chain glucosinolate level in *pad2-1* mutants. In the flavonoid biosynthetic pathway in *pad2-1* mutants, the decrease in the anthocyanin level is likely due to a reduction in the transcript level of genes encoding 2OG-Fe(II) oxygenase and PAP1 (**Figure 6B**, right). Under the control condition, the accumulation patterns of GSH and anthocyanins were roughly correlated in Arabidopsis (Xiang et al., 2001). Our results suggest that the correlation between GSH content and anthocyanin level is regulated at the transcript level.

### Identification of Key Pathways Associated with the Gene-to-Metabolite Networks Perturbed by Low Glutathione Content under Mild Abiotic Stress

In this study, we imposed mild stress conditions to obtain a better understanding of the fundamental basis of plant responses to abiotic stress. Changes in the GSH:GSSG ratio reflected their oxidative stress status under the assayed conditions (**Figure 1**). Clauw et al. (2015) who used an automated phenotyping system and genome-wide transcriptome analysis based on RNA sequencing showed that six Arabidopsis accessions exhibited common and specific response to mild drought stress (Clauw et al., 2015). Our comparative transcript profiling of the *GSH1* mutants and WT plants revealed common as well as specific stress responses, particularly against mild oxidative stress induced by a low concentration of MV and against mild stress elicited by low phosphorus (**Table 1**, **Figure 5**, and **Supplementary Figure S12**). We also evaluated the specific changes in transcript levels in response to environmental and genetic perturbations (**Figure 5**). We showed that stress-inducible genes included those encoding specific GSTs, UGTs, or CYPs that were associated with a well-known detoxification and transcriptome response when plants are exposed to oxidative stress. Our transcriptome results are in agreement with previous findings obtaining by conducting expression analysis of known “severe” oxidative stress-related genes (Dubreuil-Maurizi et al., 2011). However, we were able to find the *pad2*-specific transcript and metabolic responses under mild oxidative stress; an example is related to glucosinolate biosynthesis. The *pad2-1* mutant clearly reprograms the cellular metabolic networks and the transcriptome in response to the applied mild abiotic stress, but whether they can be reversible is unclear and needs further studies. Very severe stress causes visible symptoms in plant growth and development, for example, stunted growth, light yellow or green leaves, and rolled leaves. Such phenotypic changes generally affect metabolite profiles (for example, see Fiehn et al., 2000). In addition, we were able to see significant metabolic fluctuation even under the strictly controlled conditions (Weckwerth et al., 2004; Kusano et al., 2007a). Combined metabolite- and transcript profiling has important implications for the diagnostic characterization of the “mild” stress level and fine-tuning molecular responses in plants.

WRKY-type transcription factors play an important role in the response to biotic and abiotic stress and are also important components of a plant signaling pathway that controls developmental processes (Eulgem et al., 2000; Ulker and Somssich, 2004; Rushton et al., 2010). Interplay among three types of group I WRKY proteins, WRKY33, WRKY25, and WRKY26, is crucial for promoting heat and salt tolerance in Arabidopsis (Li et al., 2011). Arabidopsis sigma factor-binding proteins (SIB1 and SIB2) stimulate the DNA binding activity of the WRKY33 transcription factor in plant defense (Lai et al., 2011). MV treatment also induces WRKY33 expression (Zheng et al., 2006; Miller et al., 2008). Our microarray experiment showed remarkable down-regulation of WRKY33 gene expression in *pad2-1* by mild MV treatment (**Table 1**). Cross-talk between glutathione and phytohormones is also important for abiotic stress responses in Arabidopsis. Datta and colleagues showed that glutathione regulates the transcription levels of genes involved in ethylene biosynthesis via WRKY33 (Datta et al., 2015). Ethylene is known to regulate a wide range of plant processes, including growth, ripening, and responses to environmental stress (Kieber, 1997; Guo and Ecker, 2004; Klee, 2004; Yoo et al., 2009; Wang et al., 2013). As genotype-dependent altered genes and *pad2-1*-specific responsive genes, we detected changes in transcript levels of genes, including GSTs, UGTs, CYPs, HSPs, and SAGs, and transcription factors such as ERF6 and HSFA2. Our findings suggest that WRKY33 protein plays a vital role either as positive or negative transcriptional regulators, perceiving direct or indirect signal transduction in different ways under mild oxidative stress, though detailed regulatory mechanisms are still unclear.

### The Relationship between Known Stress Marker Genes and Low Glutathione Content

Although the glutathione biosynthetic pathway can be considered as a key component in the sulfur assimilation pathway (Saito, 2000, 2004; Kopriva, 2006; Takahashi et al., 2011), except for the gene encoding SUFE2 that was up-regulated in *pad2-1* compared with that in WT plants under all conditions, we observed no significant changes in the transcript level of genes involved in this pathway. Unlike our MV treatment and P-lim condition, earlier transcriptome analysis of cadmium-treated plants showed the induction of genes associated with sulfur assimilation reduction and glutathione pathways in Arabidopsis roots (Herbette et al., 2006). During oxidative stress elicited by MV and under P-lim conditions, *DREB1A*, *DREB1B*, *DREB1C*, and *DREB2A* genes were slightly up-regulated in *pad2-1*, but not in WT plants (**Table 1**). This suggests that DREB/CBF factors contribute, at least partly, to plant survival by increasing resistance under the imposed mild stress conditions. DREB2A interacts with the Radical-Induced Cell Death1 (RCD1) protein (Jaspers et al., 2010; Vainonen et al., 2012) that is involved in programmed cell death signaling and regulates plant stress responses (Overmyer et al., 2000; Ahlfors et al., 2004). The *rcd1* mutant exhibits MV and UV-B tolerance (Fujibe et al., 2004). UV-B also elicits oxidative stress, resulting in changes in photomorphogenesis, photosynthesis, membranes, and secondary metabolism (Landry et al., 1995; Mittler, 2002; Frohnmeyer and Staiger, 2003; Kusano et al., 2011). Our current understanding of the molecular mechanisms controlling programmed cell death (PCD) in plants is limited, particularly with regard to how signaling by ROS drives/regulates the changes in expression leading to PCD (Kragelund et al., 2012). These mechanisms have not yet been identified. Genes encoding ZAT12 and STZ were also up-regulated in *pad2-1* under both stress conditions (**Table 1**). ZAT12 is related to the control of cold-responsive gene expression and to the adaptation of plants to a freezing environment (Vogel et al., 2005). STZ is related to the Cys2/His2-type zinc-finger transcriptional repressor that involves drought stress tolerance (Sakamoto et al., 2004). These findings suggest that ZAT12 and STZ are also involved in plant responses to mild oxidative and low phosphorus stress.

## Conclusion

Glutathione is a critical molecule that protects plant cells against ROS and is one of the important antioxidants in abiotic stress responses in plants. Despite efforts focused on elucidating the relationship among glutathione content, biosynthesis, and abiotic/biotic stress, understanding of low glutathione-mediated plant responses to mild rather than severe oxidative and nutrient stress remains incomplete. In this study, we assessed the effects of combined low glutathione with mild oxidative and low phosphorus stress on the metabolism in Arabidopsis. This study presents integrated metabolomics and transcriptomics analysis of glutathione depletion mutants, or *GSH1* mutants (*cad2-1* and *pad2-1*), in Arabidopsis in response to mild MV-induced oxidative and low phosphate stress. The data presented here suggest that sensitivity to mild oxidative stress induced by a low concentration of MV in the mutant of the *GSH1* gene is similar to that of WT plants in terms of plant shoot growth. Our broad metabolite profiling showed that several flavonoids overaccumulated as a common oxidative stress response, whereas increased levels of flavonols with specific kaempferol- and quercetin-glycosides were observed as a common mild phosphate stress response. In addition to a significant production of sugar, osmolytes, and lipids as mild oxidative stress-responsive metabolites, we identified opposite alteration between short-chain- and long-chain aliphatic glucosinolates in the *GSH1* mutants. Genome-wide transcriptome analysis supports the metabolite responses by detecting coordinated gene expressions related to glucosinolate and flavonoid biosynthesis in the *pad2-1* mutant. We also hypothesize that *pad2-1* mutants accelerate transcriptional regulatory networks to control the biosynthetic pathways involved in glutathione-independent scavenging metabolites, and that they might reconfigure the metabolic networks in the primary and secondary metabolism of compounds, including lipids, glucosinolates, and flavonoids. The findings of our study provide a basis for the elucidation of the molecular mechanisms involved in the transcriptional regulation and reprogramming of metabolic networks in response to mild oxidative and nutrient stress in Arabidopsis.

## Availability of Supporting Data

All microarray data are available in the NCBI GEO database (Barrett et al., 2013) (accession no. GSE57286).

## Author Contributions

AF, MI, and MK conceived the project, analyzed the data, and interpreted the majority of the results. MI and TN participated in experiments on the Arabidopsis mutants, *cad2-1* and *pad2-1*, and the *35S::GSH1* transgenic lines 7-5 and 13-6, and in transcript profiling by microarrays. MK, RN, MKo, YO, and KS designed and performed metabolite profiling and drafted the Materials section. MI conducted glutathione quantification. AF, MI, and MK wrote the article with contributions from other co-authors.

## Conflict of Interest Statement

The authors declare that the research was conducted in the absence of any commercial or financial relationships that could be construed as a potential conflict of interest.

## Footnotes

1 http://www.umetrics.com

2 http://bioinfogp.cnb.csic.es/tools/venny/

3 http://www.r-project.org/

4 http://www.qiagen.com

5 http://www.affymetrix.com

6 http://www.affymetrix.com/Auth/analysis/downloads/na31/ivt/ATH1-121501.na31.annot.csv.zip

7 http://genevenn.sourceforge.net/vennresults.php

## Supplementary Material

FIGURE S1 Strategy to choose the candidate lines that showed no visible phenotypes, but had altered metabotypes compared to that of WT. See also Supplementary Table S1. **(A)** Workflow of the screening for the eight candidate lines from the 50 mutant lines. Visual phenotypes of the 50 lines were preliminary observed as described in (Fukushima et al., 2014). **(B)** PCA of metabolite profiles of the “batch 2” dataset obtained using GC-TOF-MS. Score scatter plots were shown using PC1 (13%) and PC2 (10%) in the left panel and PC1 (13%) and PC3 (8%) in the right panel, respectively.

FIGURE S2 Visible phenotypic changes in 18-day-old samples grown under different concentrations of methyl viologen (MV). We observed WT individual plants grown under Murashige and Skoog medium containing 0.01, 0.03, and 0.05 μ MV **(A)**. We also compared WT with *pad2-1* plants under different MV treatments **(B)**.

FIGURE S3 Visible phenotypic changes in 18-day-old samples grown under Murashige and Skoog medium (control MS), MS medium containing 0.05 μM of methyl viologen (MV), and phosphorous-limiting MS (P-lim, the concentration corresponds to 20% of ordinary MS medium).

FIGURE S4 Visual phenotypes, fresh weight of shoots, and GSH and GSSG levels in WT plants and *35S::GSH1*-overexpressing transgenic lines, 7-5 and 13-6, grown under three different conditions. Visible phenotypic changes in 20-day-old *GSH1*-overexpressing lines grown on Murashige and Skoog medium (no stress), Murashige and Skoog medium containing 0.05 μM methyl viologen (MV), and low-phosphate (P-lim) Murashige and Skoog medium **(A)**. Fresh weight (FW) of aerial parts of WT and transgenic plants grown under the three conditions (*n* = 8) **(B)**. Quantification of the glutathione content in WT and transgenic plants grown under the three growth conditions. Content of the reduced form of glutathione, GSH (*n* = 3, biological replicates) **(C)**. Content of the oxidized form of glutathione, GSSG (*n* = 3, biological replicates) **(D)**. GSH:GSSG ratio as an indirect determinant of oxidative stress **(E)**. Each error bar indicates the standard deviation from the mean. Asterisks represent differences from the control (significant levels were ^∗∗^ α = 0.01 and ^∗^ α = 0.05) by Welch’s *t*-test. The letters “a” and “b” represent significant differences compared to the no-stress condition (significant levels were a, α = 0.01 and b, α = 0.05) by Welch’s *t*-test. Note that the control values in glutathione levels are considerably higher than those shown in **Figure 1**, because the differences can arise due to both technical (e.g., unwanted instrumental variation) and/or biological factors (e.g., quality of the Arabidopsis seeds).

FIGURE S5 Principal component analysis of the metabolite profiles of WT plants and *GSH1* mutants (*cad2-1* and *pad2-1*) exposed to MV and P-lim; integrated data obtained from multi-platform metabolite profiling were used (*n* = 8, biological replicates). Detected metabolites contained sugar and sugar alcohols, amino acids, organic acids, fatty acids, glucosinolates, flavonoids, and lipids. The PCA score scatter plots indicate that the samples were clustered according to the corresponding genotype-dependent separation in the PC1/PC3 or PC2/PC3 direction.

FIGURE S6 Venn diagram of identified/annotated metabolites under different conditions (control, MV, and P-lim). The threshold was set at FDR < 0.05 and |log_2_fold-change|≥ 1.

FIGURE S7 Metabolite changes in the *GSH1* mutants and WT by MV and P-lim treatments. **(A)** Stress-treatment comparison: Log_2_fold-change in metabolites exposed to the two abiotic stresses. The fold-change is represented by two directed colors; red and blue indicate increase and decrease, respectively, in the metabolite level elicited by MV treatment or P-lim. **(B)** Genotype-dependent comparison: Red and blue indicate increase and decrease, respectively, in the metabolite levels in the mutants vs. WT plants. For abbreviations of the metabolite names, see Supplementary Table S5. *n* = 8, biological replicates, ^∗^FDR < 0.05.

FIGURE S8 Enrichment map showing biological processes between *pad2-1* and WT under 2 differential conditions based on hypergeometric tests of gene ontology (GO) terms by using BiNGO software (Maere et al., 2005). The map indicates the enriched GO terms in *pad2* vs. WT under MV **(A)** and under P-lim condition **(B)**. Enrichment map can be used to inspect differential transcriptomic responses between 2 genotypes (i.e., *pad2-1* vs. WT) under abiotic stress. Nodes represent GO terms and links between nodes represent gene overlap between GO terms. Inner circle size of each node represents the number of DEGs in “comparison 1” (e.g., *pad2* vs. WT under no-stress condition) within the GO term in biological process. Node border size represents the number of DEGs in “comparison 2” (e.g., *pad2-1* vs. WT under MV stress) within the GO term in biological process. Color of the node and border refer to the significance based on the BiNGO FDR of the GO term for “comparison 1” and “comparison 2,” respectively. The red-filled nodes highlight the major GO functional terms. Link size shows the number of DEGs that overlap between the two connected GO terms (Jaccard coefficient, the cut-off is 0.25). Green links correspond to both datasets when it is the only color link. Green links indicate “comparison 1” and blue indicates “comparison 2.” Blue dotted circles represent summarized GO term clusters based on AutoAnnotate (Kucera et al., 2016). The maps were generated using Cytoscape (v3.2.1) Enrichment Map plugin (Merico et al., 2010; Isserlin et al., 2014).

FIGURE S9 Overview of the transcript profiles associated with hormone metabolism based on MapMan. The Affymetrix ATH1 microarray of treatment **(A)** and genotype comparison by using MapMan software (http://mapman.gabipd.org/web/guest/mapman). The log_2_fold-change is shown by color: for **(A)** red, upregulated by stress treatment; blue, downregulated by stress treatment; for **(B)** red, upregulated in *pad2-1*; blue downregulated in *pad2-1*. A Wilcoxon test with Benjamini–Hochberg multiple-testing correction was used to identify MapMan BINs that were significant, coordinately (FDR < 0.05) compared to the others.

FIGURE S10 Overview of the transcript profiles associated with secondary metabolism based on MapMan. For detail explanation, see the legend to **Supplementary Figure S9**.

FIGURE S11 Overview of the transcript profiles associated with cell wall metabolism based on MapMan. For detail explanation, see the legend to **Supplementary Figure S9**.

FIGURE S12 Venn diagrams representing the classification of genes based on microarray data. **(A)** Down-regulated genes by MV treatment and P-lim stress. **(B)** Genotype-dependent down-regulated genes.
