# Supplementary material for: Effects of Combined Low Glutathione with Mild Oxidative and Low Phosphorus Stress on the Metabolism of Arabidopsis thaliana
Source: Front Plant Sci. 2017 Aug 28;8:1464. doi: 10.3389/fpls.2017.01464 (PMC5581396; doi:10.3389/fpls.2017.01464)
Supplement: FIGURE S1 — Strategy to choose the candidate lines that showed no visible phenotypes, but had altered metabotypes compared to that of WT. See also Supplementary Table S1. (A) Workflow of the screening for the eight candidate lines from the 50 mutant lines. Visual phenotypes of the 50 lines were preliminary observed as described in (Fukushima et al., 2014). (B) PCA of metabolite profiles of the “batch 2” dataset obtained using GC-TOF-MS. Score scatter plots were shown using PC1 (13%) and PC2 (10%) in the left panel and PC1 (13%) and PC3 (8%) in the right panel, respectively. [file Presentation_1.pdf]

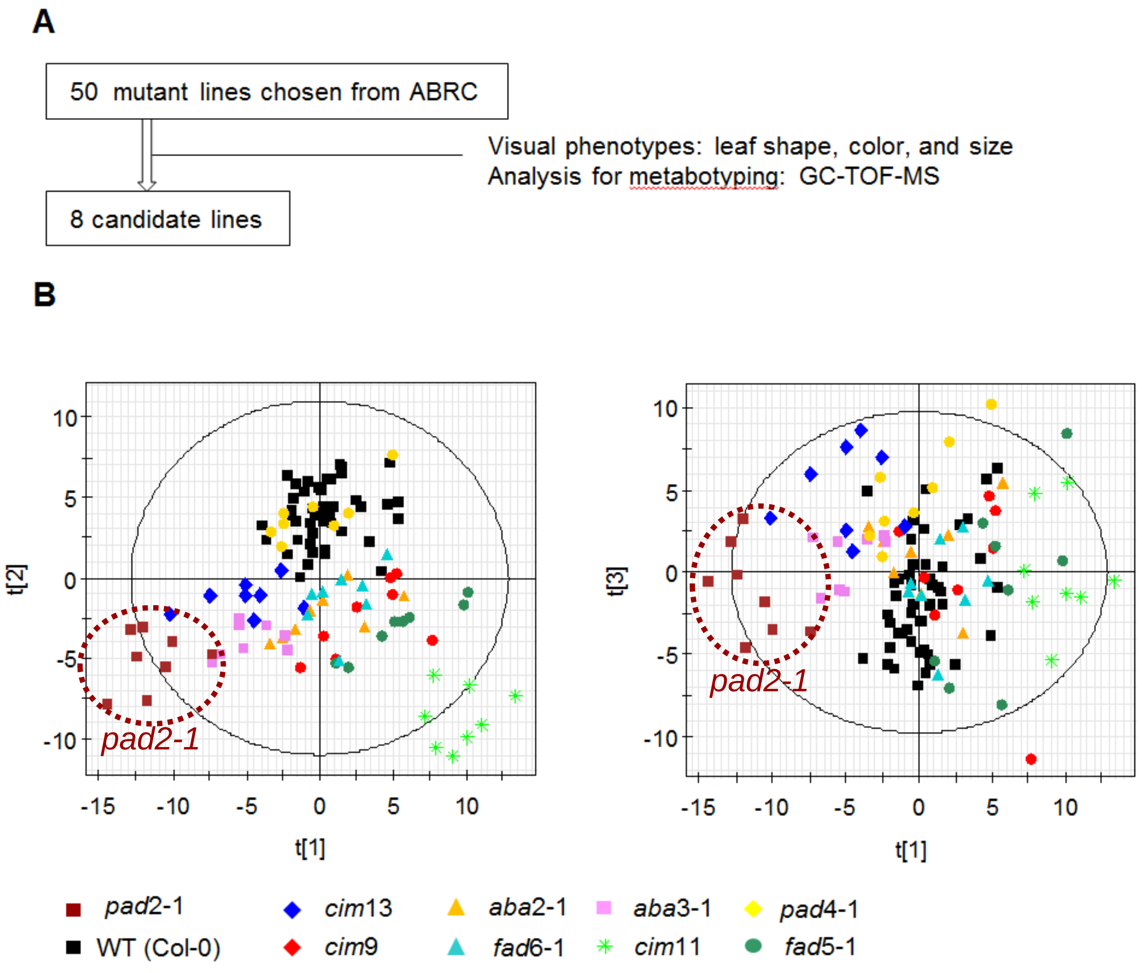

Fukushima et al., Supplemental Figure S1

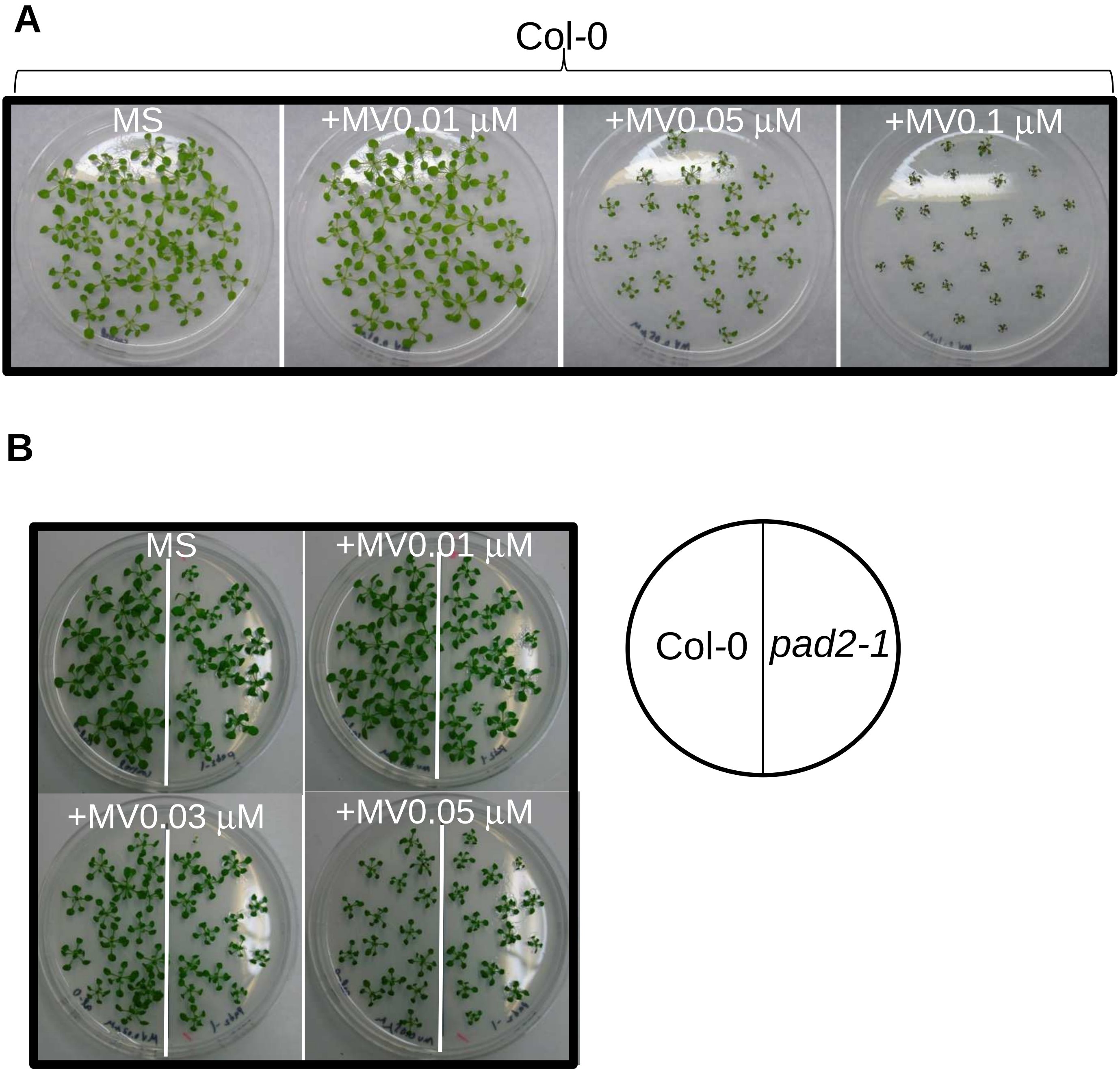

Fukushima et al., Supplemental Figure S2

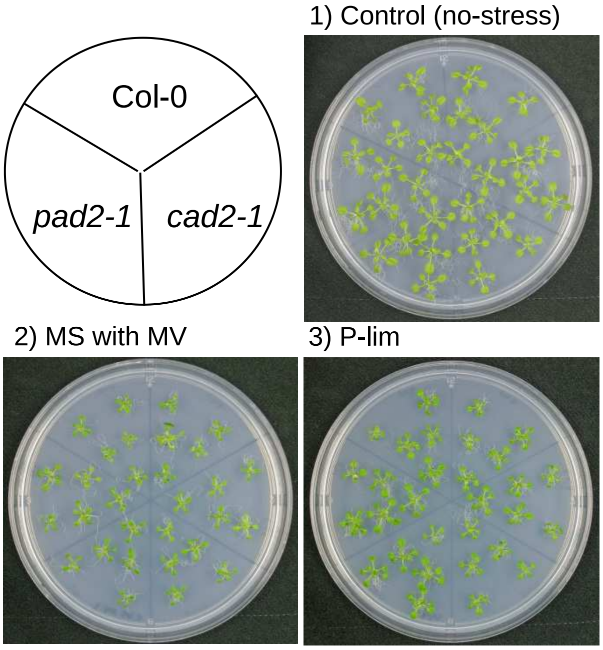

Fukushima et al., Supplemental Figure S3

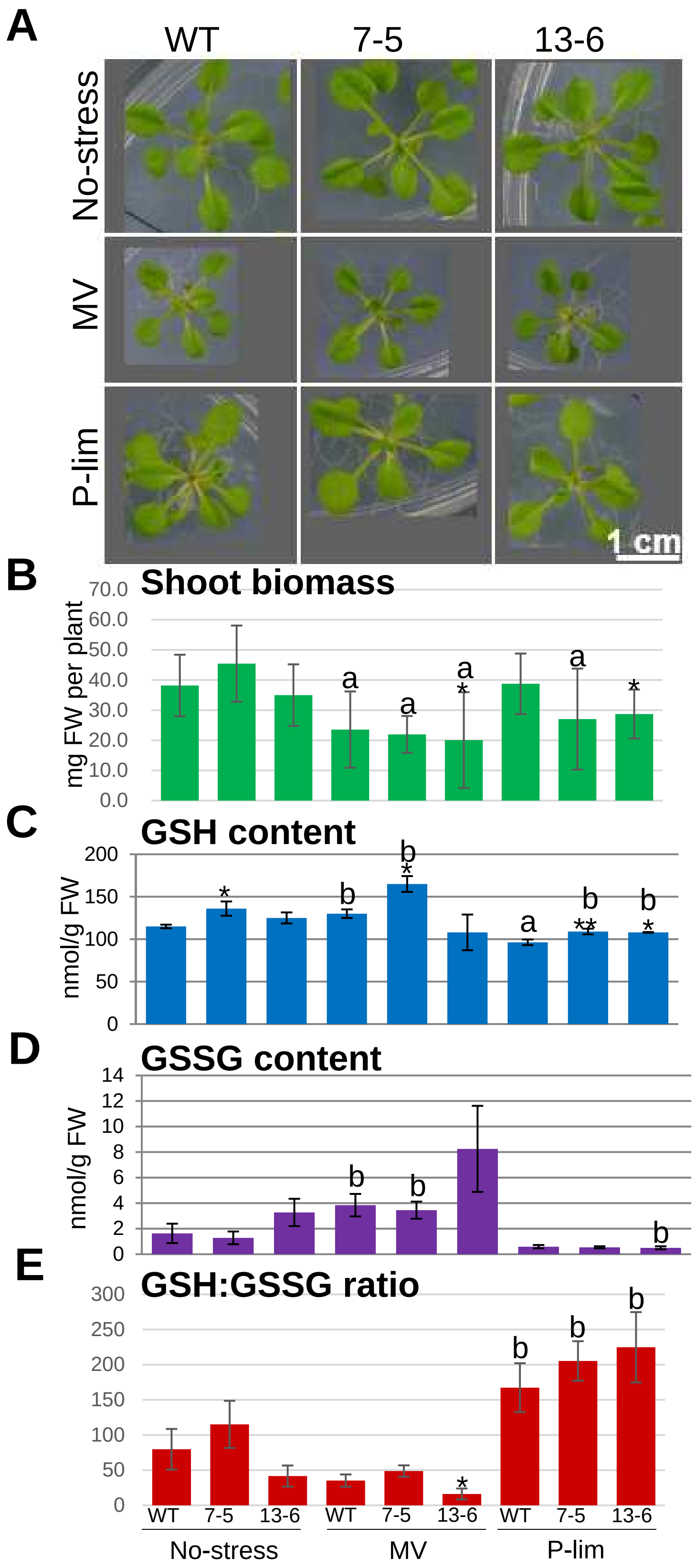

Fukushima et al., Supplemental Figure S4

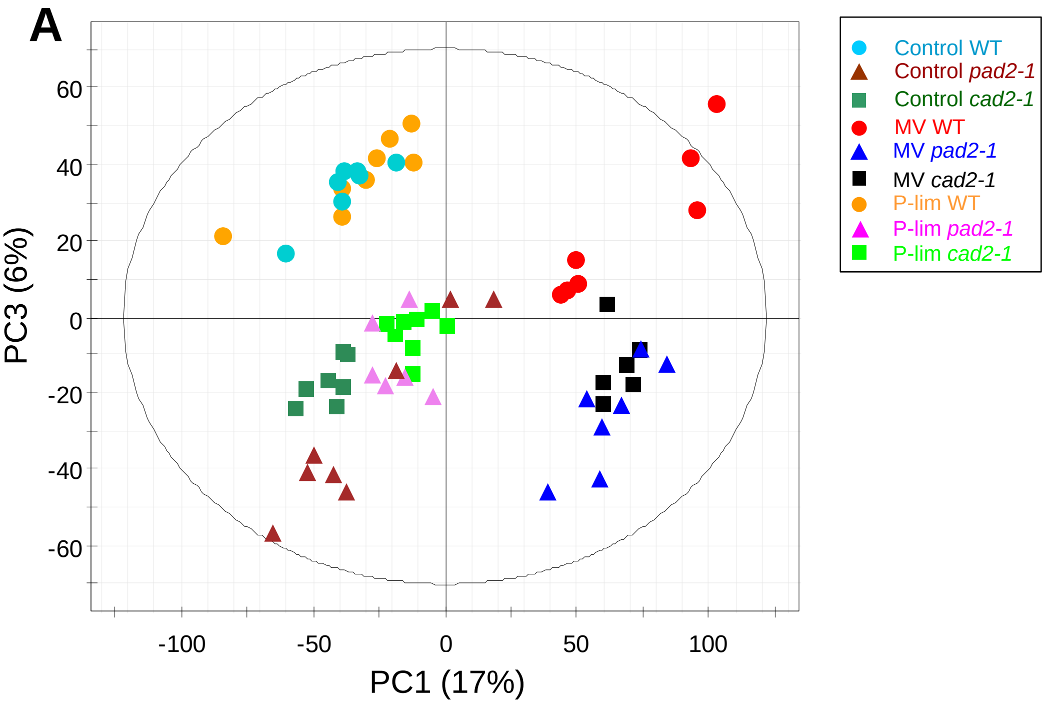

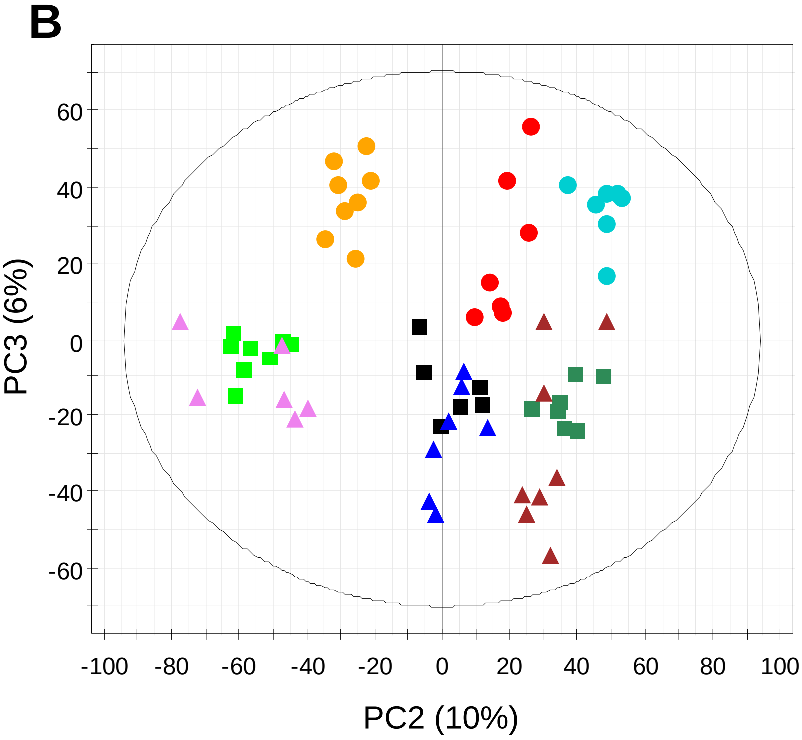

Fukushima et al., Supplemental Figure S5

A

pad2 vs WT

(FDR < 0.05, |$\log_2$fold-change| >= 1)

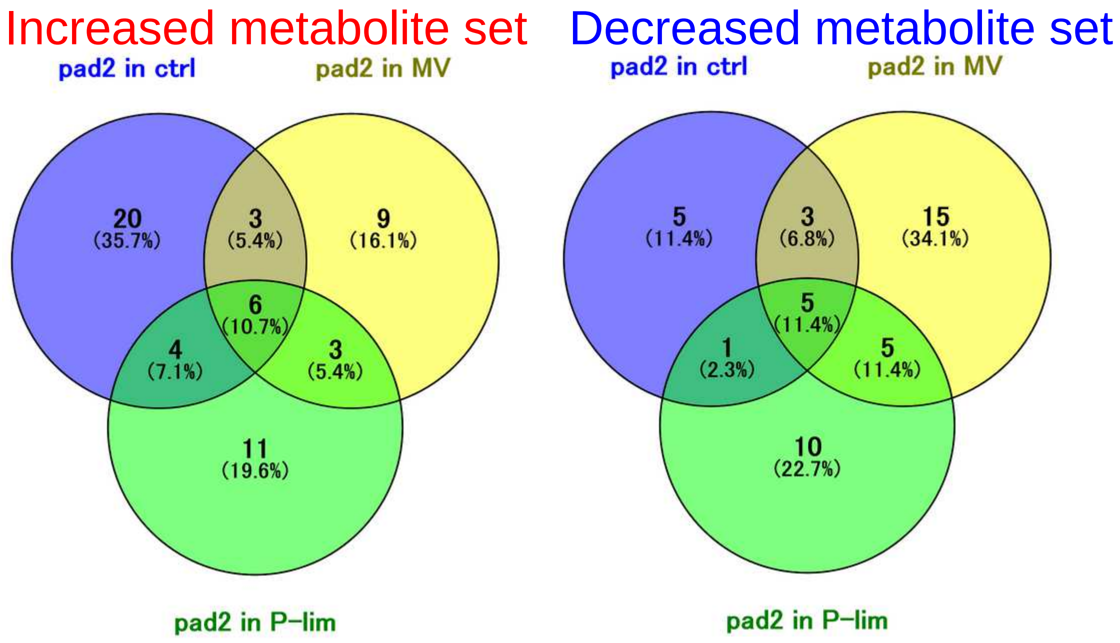

B

cad2 vs WT

(FDR < 0.05, |$\log_2$fold-change| >= 1)

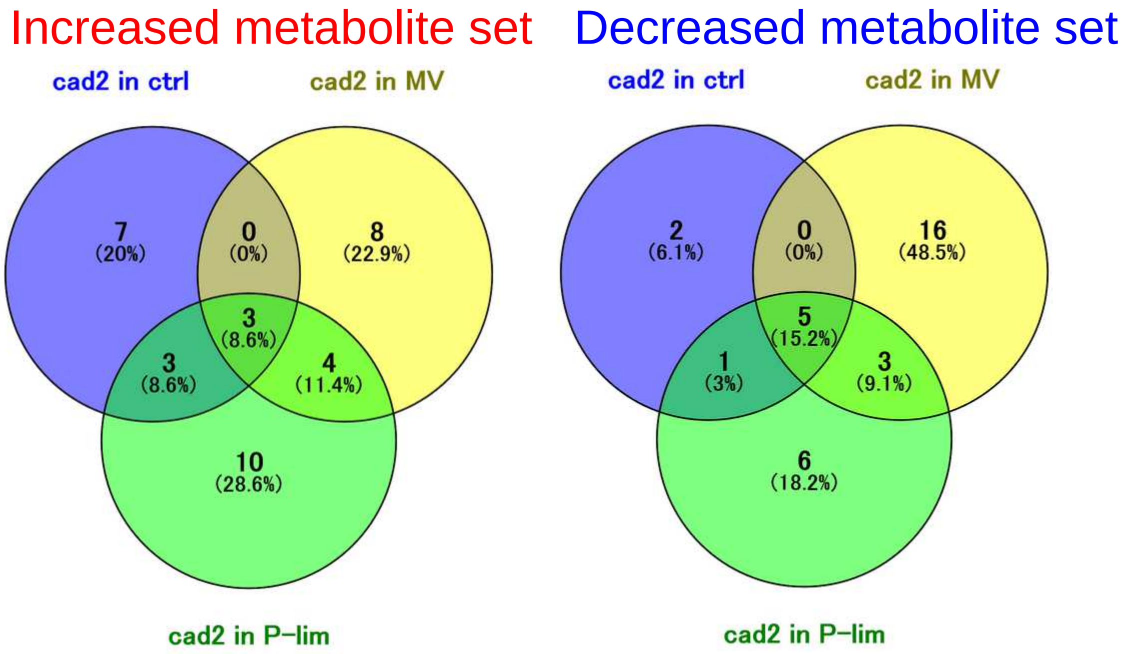

Fukushima et al., Supplemental Figure S6

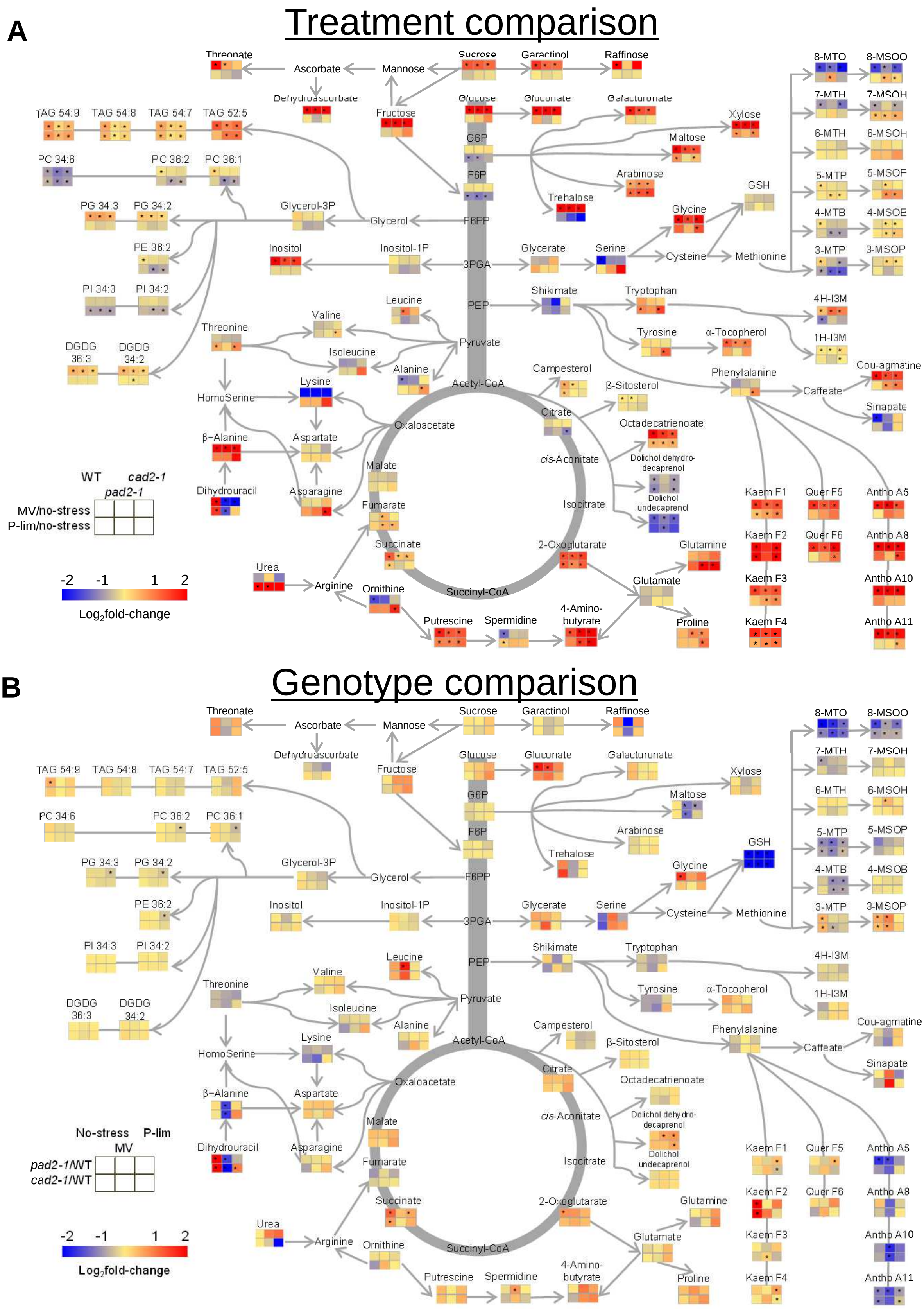

Fukushima et al., Supplemental Figure S7

A. Genotype comparison under MV

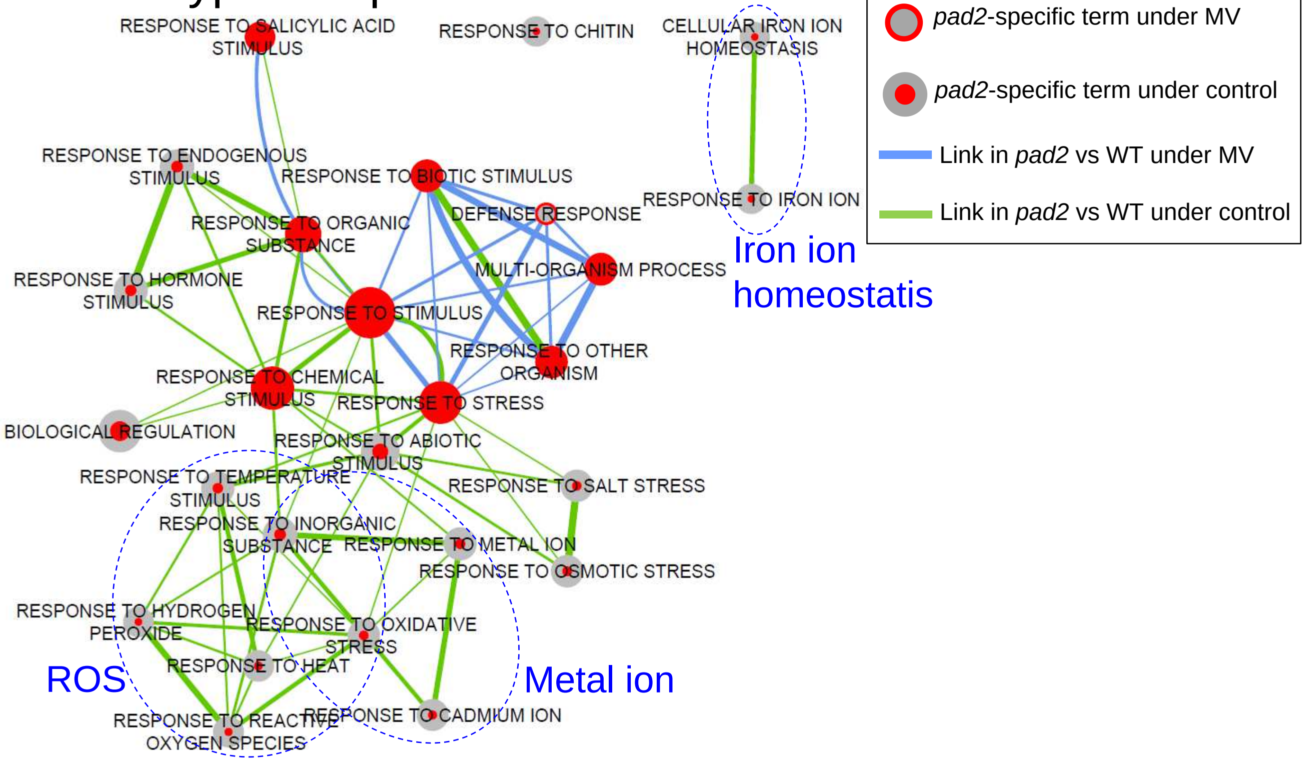

B. Genotype comparison under P-lim

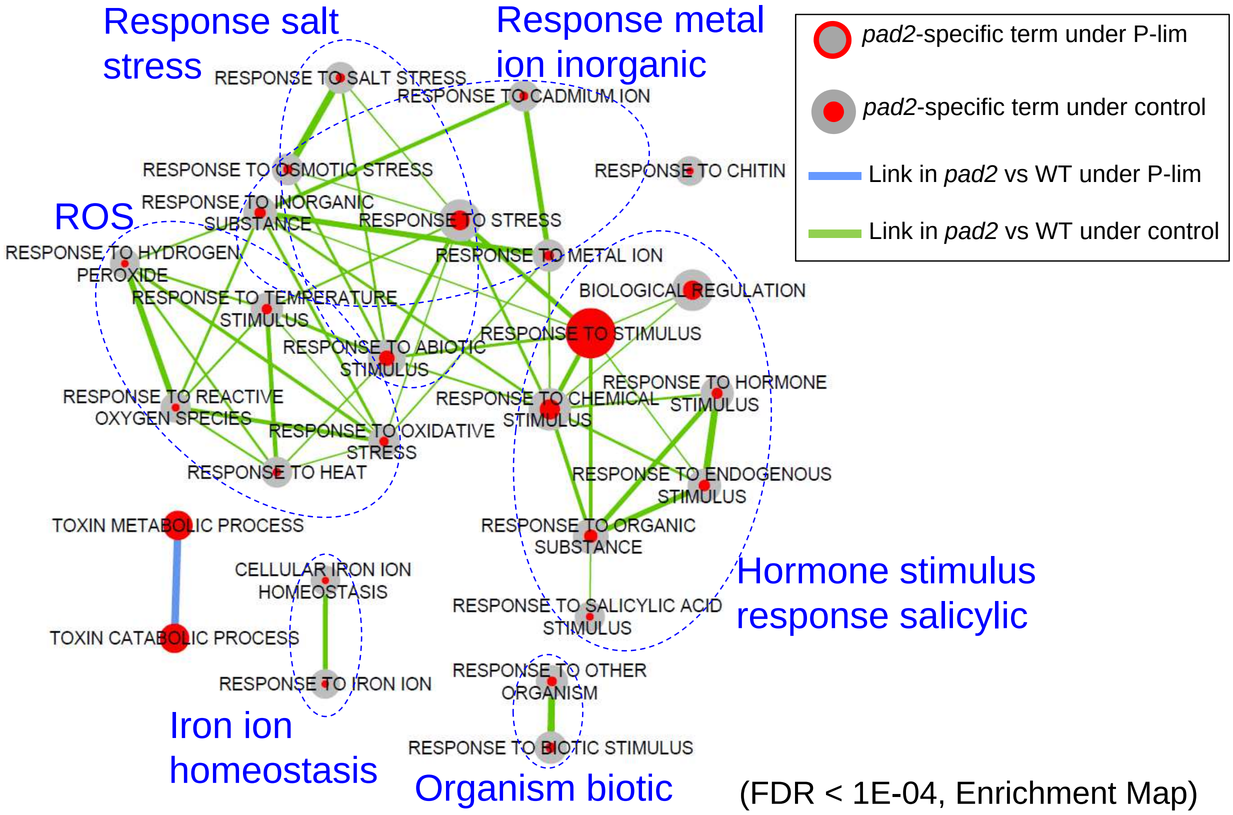

(FDR < 1E-04, Enrichment Map)

Fukushima et al., Supplemental Figure S8

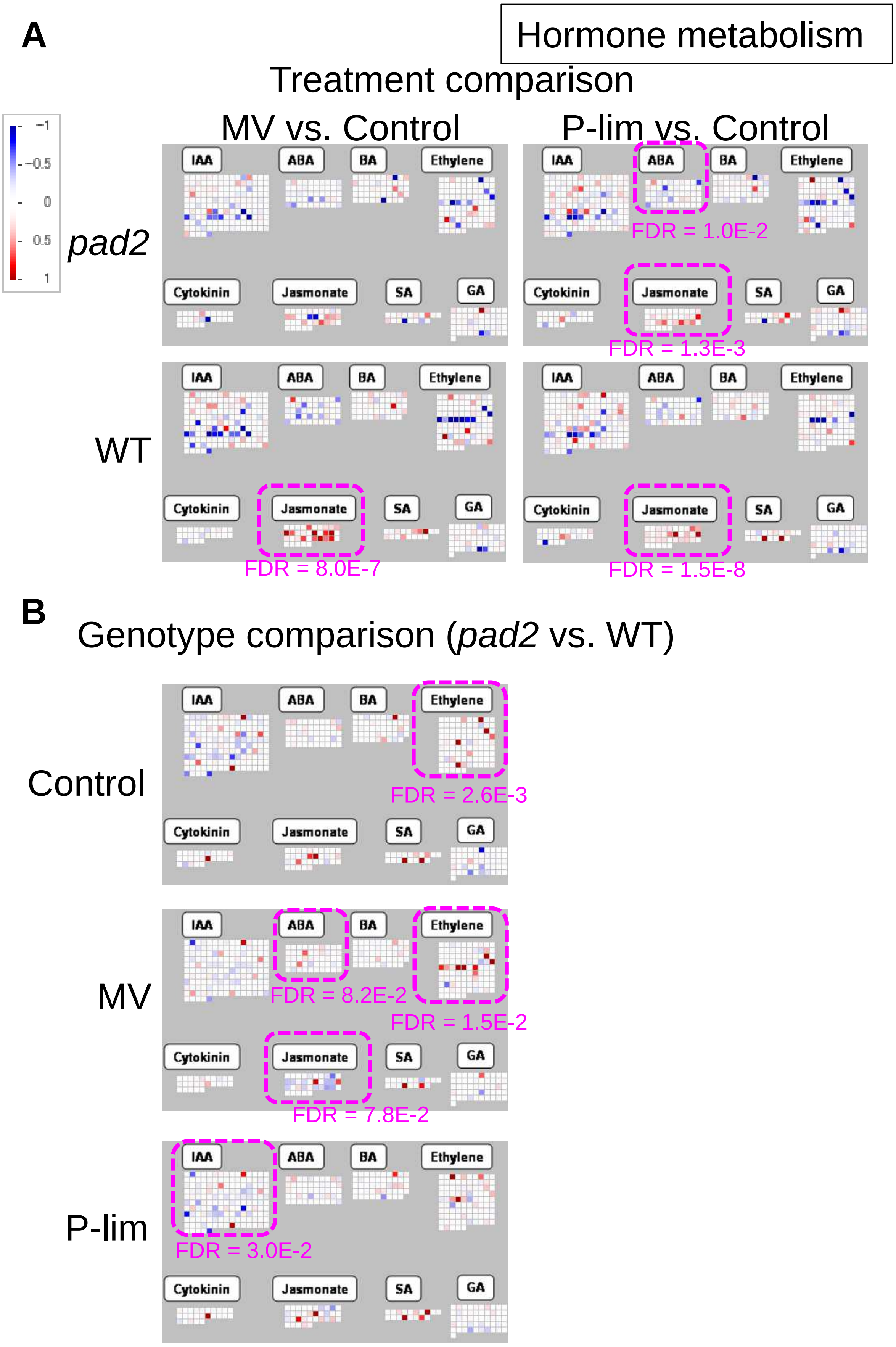

Fukushima et al., Supplemental Figure S9

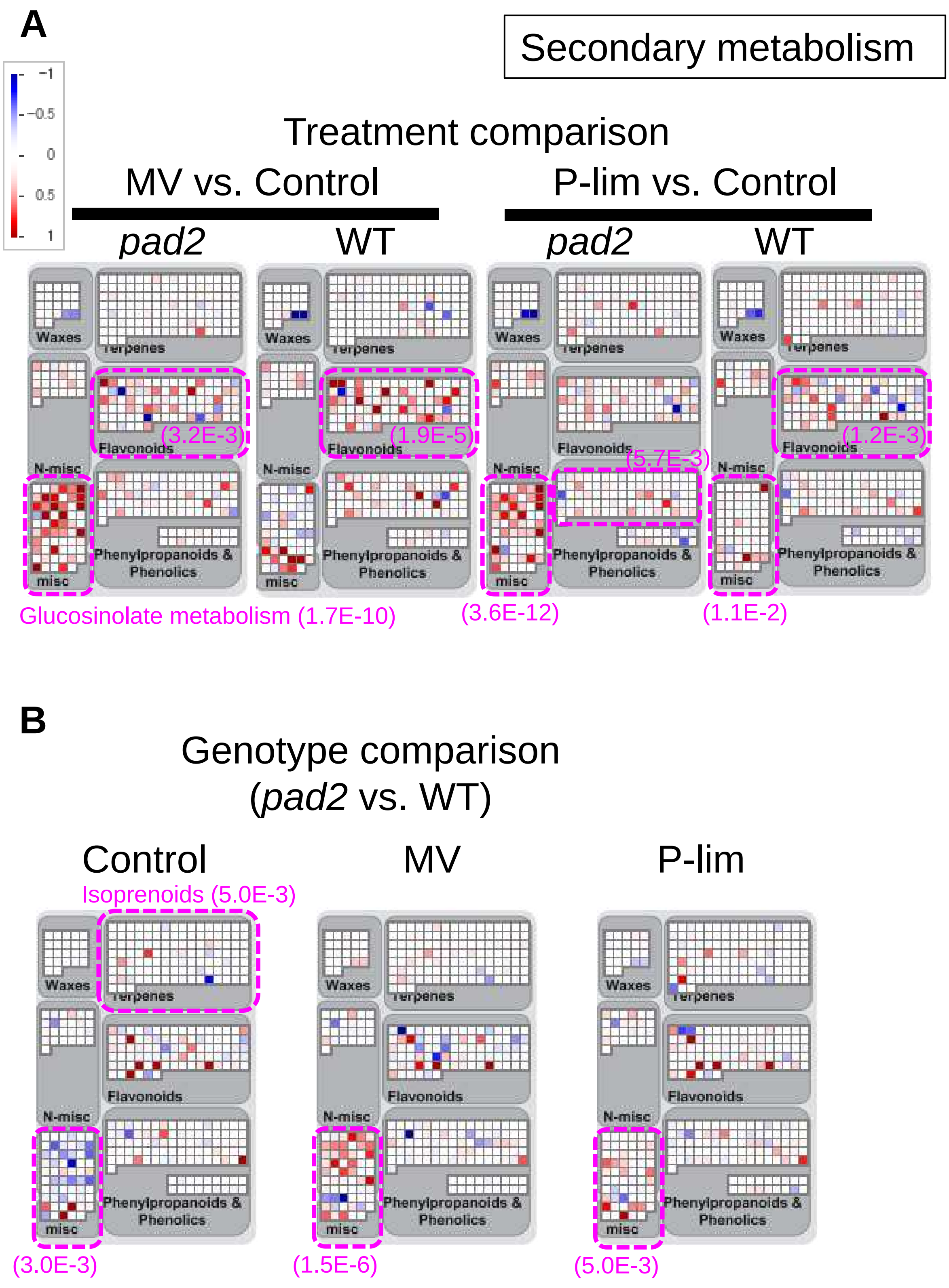

Fukushima et al., Supplemental Figure S10

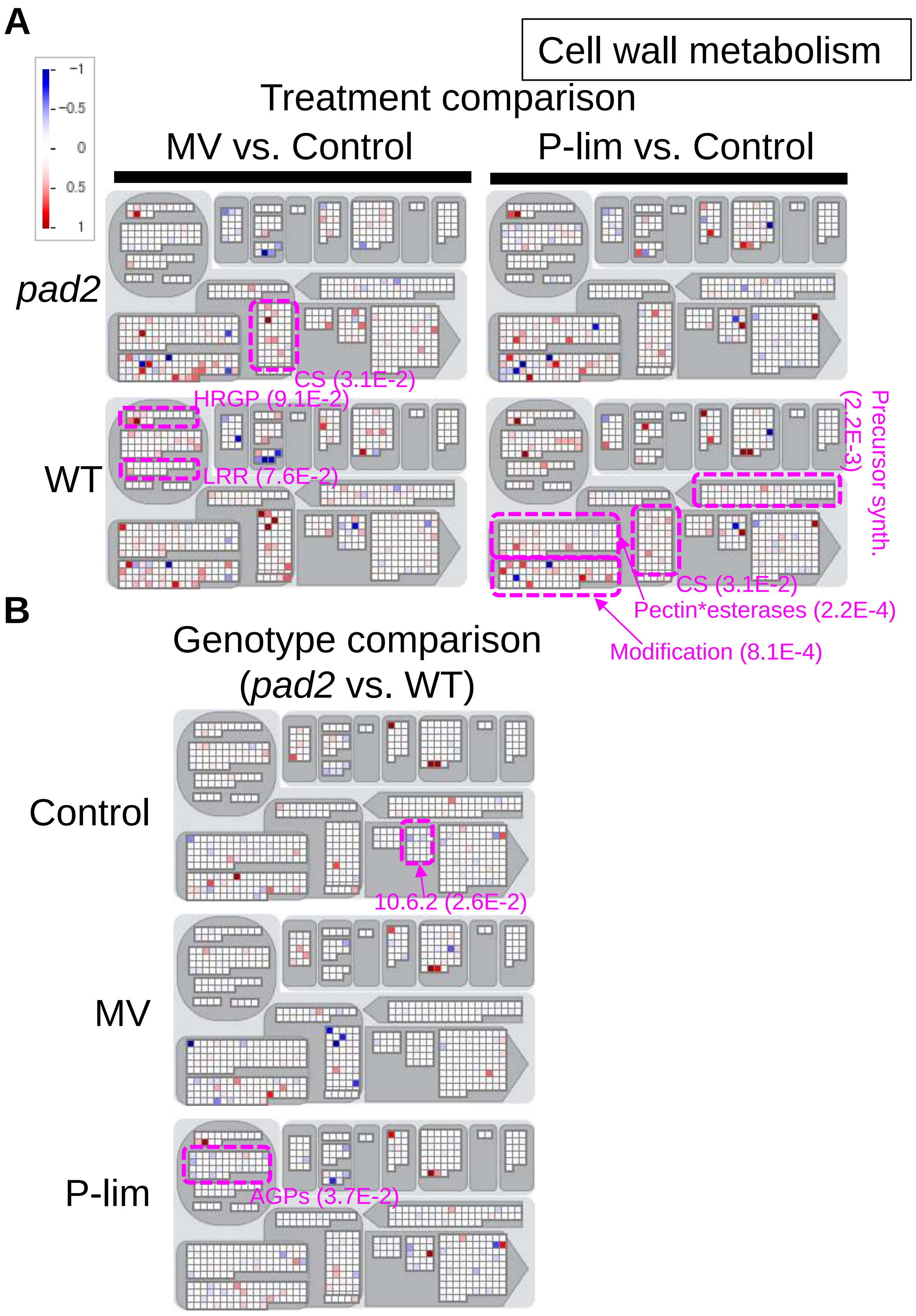

Fukushima et al., Supplemental Figure S11

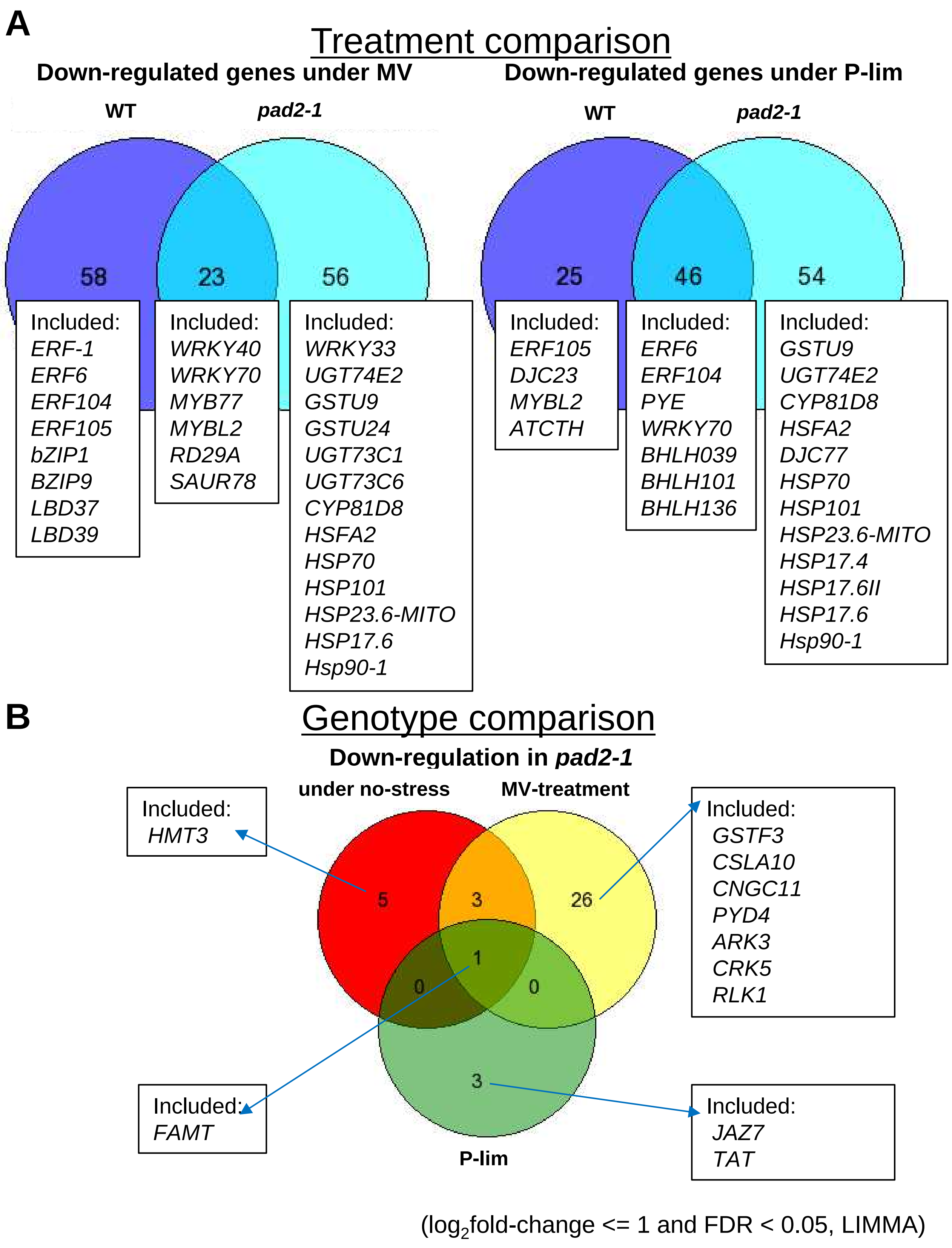

Fukushima et al., Supplemental Figure S12
