# Supplementary material for: Effects of Combined Low Glutathione with Mild Oxidative and Low Phosphorus Stress on the Metabolism of Arabidopsis thaliana
Source: Front Plant Sci. 2017 Aug 28;8:1464. doi: 10.3389/fpls.2017.01464 (PMC5581396; doi:10.3389/fpls.2017.01464)
Supplement: Supplementary file 7 [file Data_Sheet_6.DOCX]

Supplementary Material

Effects of Combined Low Glutathione with Mild Oxidative and Low Phosphorus Stress on the Metabolism of *Arabidopsis thaliana*

Atsushi Fukushima^1,†^, Mami Iwasa^1,2,†^, Ryo Nakabayashi^1^, Makoto Kobayashi^1^, Tomoko Nishizawa^1^, Yozo Okazaki^1^, Kazuki Saito^1,3^, Miyako Kusano^1,4,*^

**^†^These authors contributed equally to this work**

*** Correspondence:** Miyako Kusano, [kusano.miyako.fp@u.tsukuba.ac.jp](mailto:kusano.miyako.fp@u.tsukuba.ac.jp)

**Supplemental Document S1. Metabolomics Metadata**

# Plant context metadata

- 1. **Plant materials**
     1. **BioSource Species**

*Arabidopsis thaliana*

- - 1. ***Genotypes/Varieties***

Columbia (Col-0), *pad2-1*, and *cad2-1*

- - 1. ***Organ specification***

Aerial part

- - 1. ***Growth conditions***

Plants were grown in MS agar medium except for the oxidative and phosphorous limited condition. Sterilized seeds were stratified at 5°C for 2 days, and were sown on Murashige and Skoog (MS) medium containing 1% sucrose (Control). Oxidative and Phosphorous limited conditions were produced adding 0.05 μM Methyl Viologen and reducing phosphate concentration to 1/5 of MS medium. Seedlings of Arabidopsis Col-0 and the mutants were cultivated in growth chambers at 22°C in the 16-h light and 8-h dark condition for 18 days (light strength, 80 μmol m^-2^ s^-1^ of photosynthetic photon flux (PPF)). We sampled 20 independent plants (*n* = 20, biological replicates) for measurement of shoot biomass (flesh weight), 8 for metabolite profiling, and 3 for absolute glutathione quantification as follows.

1. **Chemical analysis metadata**

***Chemicals***

All the chemicals and reagents that were used for this study were of spectrometric grade. Chemicals excluding isotope reference compounds and reagents for silylation were purchased from Sigma Aldrich (Tokyo, Japan), NacalaiTesque (Kyoto, Japan), or Wako Pure Chemical Industries (Osaka, Japan). The 6 stable isotope compounds ([^13^C_5_]-proline, [^2^H_4_]-succinic acid, [^2^H_6_]-2-hydroxybenzoic acid, [^13^C_3_]-myristic acid, [^13^C_12_]-sucrose, and [^2^H_7_]-cholesterol) were purchased from Cambridge Isotope Laboratories (Andover, MA, USA); [^13^C_5_,^15^N]-glutamic acid and [^13^C_6_]-glucose from Spectra Stable Isotopes (Columbia, Maryland, USA), [^2^H_4_]-1,4-diaminobutane was from C/D/N ISOTOPES (Pointe-Claire, Quebec, Canada), and [^13^C_4_]-hexadecanoic acid from Icon (Mt. Marion, NY, USA). The reagent for trimethylsilylation, *N*-methyl-*N*-trimethylsilyltrifluoroacetamide (MSTFA) was purchased from Tokyo Chemical Industry (Tokyo, Japan).

- 1. ***Sample processing and extraction***
     1. ***Extraction and derivatization for GC-TOF-MS***

Each frozen sample with a 5-mm zirconia bead was extracted with 40 fold amount of solvent (methanol/chloroform/water [3:1:1 v/v/v]) containing 10 stable isotope reference compounds at 4°C in a mixer mill (MM301; Retsch, Haan, Germany) at a frequency of 15 Hz. Each isotope compound was adjusted to a final concentration of 15 ngper 1-μl injection volume. After 5-min centrifugation at 15,100 × g, a 200-μl aliquot of the supernatant was transferred to a glass insert vial. The extracts were evaporated to dryness in an SPD2010 SpeedVac® concentrator (Thermo Fisher, Scientific, Waltham, MA, USA). We used extracts from 5-mg FW samples for derivatization, i.e., methoxymation and silylation. For methoxymation, 30 μl of methoxyamine hydrochloride (20 mg/ml in pyridine) were added to the sample. After 17 h of derivatization at room temperature the sample was trimethylsilylated for 1 h using 30 µl of MSTFA at 37°C with shaking. All derivatization steps were performed in a vacuum glove box VSC-100 (Sanplatec, Osaka, Japan) filled with 99.9995% (G3 grade) dry nitrogen.

- - 1. ***Extraction for LC-q-TOF-MS to detect secondary metabolites***

Each frozen sample was extracted with5 fold amount of solvent (methanol/water [8:2 v/v]) containing reference compounds (0.5 mg/L of lidocaine ([M+H]^+^, *m/z* 235.1804) and 10-camphorsulfonic acid ([M-H]^-^, *m/z* 231.0691) using a mixer mill MM301 (Retsch) at a frequency of 20 Hz for 5 min at 4°C. After centrifugation for 10 min at 15,000 × g, the supernatant was transferred into a 2 ml tube. Aliquot of the extracts was filtered using an Oasis® HLB μelusion plate (30 μm, Waters Co., Massachusetts, US). The extracts were transferred into a 2 ml tube.

- - 1. ***Extraction for LC-q-TOF-MS to detect lipids***

Each frozen samplewas milled using mixer mill MM301 (Retsch) at a frequency of 20 Hz for 2 min at 4°C. After that, frozen powder was extracted with20 fold volume of extraction solvent (chloroform/methanol/waer[50 : 100 : 31.45, v/v])containing 0.25mM of 1,2-dioctanoyl-sn-glycero-3-phosphocholine (SIGMA). Samples were vigorously mixed using a vortex mixture. 52.5 μl of water and 52.5 μl of chloroform were added to 200 μlof extract and then vigorously mixed for 5 min at room temperature. After standing for 15 min on ice, the samples were centrifuged at 1,000 ×*g* at 5°C for 5 min. The supernatant (85 μl) was transferred to a 2 ml tube with insert. Each extract was evaporated to dryness by SPD2010 SpeedVac® concentrator (Thermo Fisher Scientific). The residue was dissolved in 162μl of ethanol, and centrifuged at 10,000×*g* at 45°C for 15 min. Two hundred microliter of the supernatant was transferred to a glass tube for lipid analysis.

- 1. ***Analyticalconditions***
     1. ***GC-TOF-MS conditions***

Using the splitless mode of a CTC CombiPALautosampler (CTC Analytics, Zwingen, Switzerland), 1 μl of each sample (equivalent to 1.4 µg DW) was injected into an Agilent 6890N gas chromatograph (Agilent Technologies, Wilmingston, DE, USA) featuring a 30 m × 0.25 mm inner diameter fused-silica capillary column and a chemically bound 0.25-μl ﬁlm Rxi-5 Sil MS stationary phase (RESTEK, Bellefonte, PA, USA) with a tandem connection to a fused silica tube (1 m, 0.15 mm). An MS column change interface (msNoVent-J; SGE, Yokohama, Japan) was used to prevent air and water from entering the MS during column change-over. Helium was the carrier gas at a constant ﬂow rate of 1 ml min^-1^. The temperature program for GC-MS analysis started with a 2-min isothermal step at 80°C followed by 30°C temperature-ramping to a ﬁnal temperature of 320°C that was maintained for 3.5 min. The transfer line and the ion source temperatures were 250 and 200°C, respectively. Ions were generated by a 70-eV electron beam at an ionization current of 2.0 mA. The acceleration voltage was turned on after a solvent delay of 222 sec. Data acquisition was on a Pegasus IV TOF mass spectrometer (LECO, St. Joseph, MI, USA); the acquisition rate was 30 spectras^-1^ in the mass range of a mass-to-charge ratio of m/z = 60–800.

Alkane standard mixtures (C8 - C20 and C21 - C40) purchased from Sigma-Aldrich (Tokyo, Japan) were used for calculating the retention index (RI) ([Schauer et al., 2005](#_ENREF_5)). For quality control we injected methylstearate into every 6th sample. The sample run order was randomized in single-sequence analyses. We analyzed the standard compound mixtures using the same sequence analysis procedures.

- - 1. ***LC-q-TOF-MS conditions to detect secondary metabolites***

After preparation of the extracts, the sample extracts (1 μl) were analyzed using an LC-MS system equipped with an electrospray ionization (ESI) interface (LC, Waters Acquity UPLC system; MS, Waters Xevo G2 Q-Tof). The analytical conditions were as follows. LC: column, Acquity bridged ethyl hybrid (BEH) C18 (pore size 1.7 μm, length 2.1× 100 mm, Waters); solvent system,acetonitrile(0.1% formic acid):water (0.1% formic acid); gradient program,

1 : 99 v/v at 0 min, 1 : 99 v/v at 0.1 min, 99.5 : 0.5 at 15.5 min,99.5 : 0.5 at 17.0 min, 1 : 99 v/v at 17.1 min and 1 : 99 at 20 min, flow rate, 0.3 ml/min,temperature, 40°C; MS detection: capillary voltage, +3.0 keV, cone voltage, 25.0 V, source temperature, 120°C, desolvation temperature, 450°C, cone gas flow, 50 l per h; desolvation gas flow, 800 l per h; collision energy, 6 V; mass range, *m/z* 100‒1500; scan duration, 0.1 sec; interscan delay, 0.014 sec; mode, centroid; polarity, positive; Lockspray (Leucineenkephalin): scan duration, 1.0 sec; interscan delay, 0.1 sec. The data were recorded using MassLynx version 4.1 software (Waters).

- - 1. ***LC-q-TOF-MS conditions to detect lipids***

Sample extracts (1 μl) were analyzed using an LC-MS system equipped with an electrospray ionization (ESI) interface (HPLC, Waters Acquity UPLC system; MS, Waters Xevo G2 Qtof). Two-solvent (A and B) system was used for separation of each metabolite. Compositions of these solvents were as follows: solvent A, acetonitrile: water:1 M ammonium acetate:formic acid = (158 g:800g:10 ml:1 ml); solvent B, acetonitrile:2-propanol:water:1 M ammonium acetate:formic acid = (79 g:711 g:10 ml:1 ml). The analytical conditions were as follows. HPLC: column, Acquity UPLC HSS T3 (pore size 1.8 μm, 1.0 i.d × 50 mm long, Waters); gradient program, 35% B at 0 min, 70% B at 3 min, 85% B at 7 min, 90% B at 10 min, 90% B at 12 min and 35% B at 12.5 min; flow rate, 0.15 ml/min; temperature, 55°C; MS detection: capillary voltage, +3.0 kV; cone voltage, 20 V for positive mode and 40 V for negative mode; source temperature, 120°C; desolvation temperature, 450°C; cone gas flow, 50 l/h; desolvation gas flow, 450 l/h; collision energy, 6 V; detection mode, scan (*m/z* 100–2000; scan time, 0. 5 sec; centroid). The scans were repeated for 15 min in a single run. The data were recorded using MassLynx version 4.1 software (Waters).

- 1. ***Data processing***
     1. ***Data processing for GC-TOF-MS data***

Nonprocessed MS data from GC-TOF-MS analysis were exported in NetCDF format generated by chromatography processing- and mass spectral deconvolution software (LecoChromaTOF version 3.22; LECO, St. Joseph, MI, USA) to MATLAB 6.5 or MATLAB2011b (Mathworks, Natick, MA, USA) for the performance of all data-pretreatment procedures, e.g. smoothing, alignment, time-window setting H-MCR, and RDA ([Jonsson et al., 2006](#_ENREF_1)). The resolved MS spectra were matched against reference mass spectra using the NIST mass spectral search program for the NIST/EPA/NIH mass spectral library (version 2.0) and our custom software for peak-annotation written in JAVA. Peaks were identified or annotated based on their RIs, a comparison of the reference mass-spectra with the GolmMetabolome Database (GMD) released from CSB.DB ([Kopka et al., 2005](#_ENREF_2)), and our in-house spectral library. The metabolites were identified by comparison with RIs from the library databases (GMD and our own library) and the RIs of authentic standards. The metabolites were defined as annotated metabolites after comparison with the mass spectra and the RIs from these two libraries. The data matrix was normalized using the CCMN algorithm for further analysis ([Redestig et al., 2009](#_ENREF_3)) .

- - 1. ***Data processing for LC-q-TOF-MS data to detect secondary metabolites***

The data matrix was aligned by MassLynx version 4.1 (Waters). The profiling data files were converted to the NetCDF format using the DataBridge function of the MassLynx software. The data matrices were processed using in-house Perl script for alignment and deisotope with the set of NetCDF data files.

For normalization, intensity values of remained peaks was divided by those of the lidocaine ([M+H]^+^, *m/z* 235.1804) and 10-camphorsulfonic acid ([M-H]^-^, *m/z* 231.0691) after cutoff of the low-intensity peaks (less than 500 counts).

- - 1. ***Data processing for LC-q-TOF-MS data to detect lipids***

The data matrix was generated using the Makerlynx XS (Waters) using the profiling data files recorded in the MassLynx format (raw). The data matrices were processed using in-house Perl script. The original peak intensity values were divided with that of the internal standard (didecanoyl-sn-glycerophosphocholine at *m/z* 566.382 [M + H]^+^ and at *m/z* 610.372 [M + HCOO]^–^ for the positive and negative ion modes, respectively) to normalize the peak intensity values among the metabolic profile data.

- 1. ***Statistical data analysis for metabolite profile data***

The multi-platform data was summarized by unifying metabolite identifiers to a common referencing scheme using the MetMask tool ([Redestig et al., 2010](#_ENREF_4)). The four matrices were then concatenated and correlated peaks with the same annotation were replaced by their first principal component. All data was log_10_ transformed prior to further data analysis. Principal component analysis (PCA) was performed on unit-variance scaled metabolite matrixes (observations, 81 samples; variables, 681 or 701 peaks) with log_10_ transformation using the pcaMethods package ([Stacklies et al., 2007](#_ENREF_6)) or SIMCA-P+ 12.0 software (Umetrics AB, Umeå, Sweden).

**References**

**Jonsson P, Johansson ES, Wuolikainen A, Lindberg J, Schuppe-Koistinen I, Kusano M, Sjostrom M, Trygg J, Moritz T, Antti H** (2006) Predictive metabolite profiling applying hierarchical multivariate curve resolution to GC-MS data--a potential tool for multi-parametric diagnosis. J Proteome Res **5:** 1407-1414

**Kopka J, Schauer N, Krueger S, Birkemeyer C, Usadel B, Bergmuller E, Dormann P, Weckwerth W, Gibon Y, Stitt M, Willmitzer L, Fernie AR, Steinhauser D** (2005) GMD@CSB.DB: the Golm Metabolome Database. Bioinformatics **21:** 1635-1638

**Redestig H, Fukushima A, Stenlund H, Moritz T, Arita M, Saito K, Kusano M** (2009) Compensation for systematic cross-contribution improves normalization of mass spectrometry based metabolomics data. Anal Chem **81:** 7974-7980

**Redestig H, Kusano M, Fukushima A, Matsuda F, Saito K, Arita M** (2010) Consolidating metabolite identifiers to enable contextual and multi-platform metabolomics data analysis. BMC Bioinformatics **11:** 214

**Schauer N, Steinhauser D, Strelkov S, Schomburg D, Allison G, Moritz T, Lundgren K, Roessner-Tunali U, Forbes MG, Willmitzer L, Fernie AR, Kopka J** (2005) GC-MS libraries for the rapid identification of metabolites in complex biological samples. FEBS Lett **579:** 1332-1337

**Stacklies W, Redestig H, Scholz M, Walther D, Selbig J** (2007) pcaMethods--a bioconductor package providing PCA methods for incomplete data. Bioinformatics **23:** 1164-1167
